# Pediatric Sleep Tools: An Updated Literature Review

**DOI:** 10.3389/fpsyt.2020.00317

**Published:** 2020-04-23

**Authors:** Tabitha Sen, Karen Spruyt

**Affiliations:** ^1^ School of Biomedical Sciences, Queensland University of Technology, Brisbane, QLD, Australia; ^2^ Lyon Neuroscience Research Center, INSERM U1028-CNRS UMR 5292, University Claude Bernard, School of Medicine, Lyon, France

**Keywords:** sleep duration, sleep quality, sleep hygiene, questionnaire, child, review

## Abstract

Since a thorough review in 2011 by Spruyt, into the integral pitfalls of pediatric questionnaires in sleep, sleep researchers worldwide have further evaluated many existing tools. This systematic review aims to comprehensively evaluate and summarize the tools currently in circulation and provide recommendations for potential evolving avenues of pediatric sleep interest. 144 “tool”-studies (70 tools) have been published aiming at investigating sleep in primarily 6–18 years old per parental report. Although 27 new tools were discovered, most of the studies translated or evaluated the psychometric properties of existing tools. Some form of normative values has been established in 18 studies. More than half of the tools queried general sleep problems. Extra efforts in tool development are still needed for tools that assess children outside the 6-to-12-year-old age range, as well as for tools examining sleep-related aspects beyond sleep problems/disorders. Especially assessing the validity of tools has been pursued vis-à-vis fulfillment of psychometric criteria. While the Spruyt et al. review provided a rigorous step-by-step guide into the development and validation of such tools, a pattern of steps continue to be overlooked. As these instruments are potentially valuable in assisting in the development of a clinical diagnosis into pediatric sleep pathologies, it is required that while they are primary subjective measures, they behave as objective measures. More tools for specific populations (e.g., in terms of ages, developmental disabilities, and sleep pathologies) are still needed.

## Introduction

There is significant power in the efficiency and cost-effective nature of questionnaires and surveys as contributors to aetiological discoveries of a wide range of medical disorders. These instruments however, do not always possess the objective nature of medically advised and established tools, e.g., polysomnography, and can become a hindrance to adequate diagnoses, particularly when neglecting recommendations of their development (1). Despite these problems, there has been considerable effort to transform the structure of health questionnaires, specifically in the field of pediatric sleep, to reflect a systematic approach of the highest concordance to medical diagnostic standards. The systematic review by Spruyt et al. (2, 3) in 2011, publicly summarized the shortcomings of questionnaires and their developmental standards while advising a thorough procedure in which to follow to adequately evaluate or develop a tool.

Since this time, a variety of tools have been established, both adhering to and overlooking the recommended steps. More detailed information on the 11 steps can be found in Spruyt et al. (3). Briefly, *Step 1* is to reflect on the variable(s) of interest and targeted sample(s). *Step 2* is to consider the research question that the instrument will be used to address. Thus, the goal of this step is to reflect on whether the tool will be suitable to collect the type of data required to address your hypothesis. *Steps 3* (response format) and *Step 4* (items) build on the two preceding steps. They allow us to reflect not only on “which” questions and “which’” answers assesses the variable(s) of interest, but also on “how” a question is formulated and “how” it can be answered. The common goal of steps 1–4 is that we want the underlying “concepts” and/or “assumptions” contained in the questions, such as language (e.g., jargon), meaning and interpretation of the wording to be identically understood by all respondents. Getting as close as this ideal as possible will minimize errors of comprehension and completion. *Step 5* involves piloting of your drafted tools. Piloting also prevents disasters with the actual data collection. In fact, *Steps 2–5* should be an iterative process, meaning that we do them repeatedly, until a consensus has been reached among experts and/or respondents with descriptive statistics underpinning those decisions. Assessing the performance of individual test items, separately and as a whole, is *Step 6* (item analysis). There are two main approaches to item analysis: classical test theory and the item-response theory, either of which should be combined with missing data analysis. The next step is about identifying the underlying concepts of the tool (*Step 7* Structure) because only rarely is a questionnaire unidimensional. *Steps 8* and *9* are about assessing the reliability and validity, respectively. Reliability does not imply validity, although a tool cannot be considered valid if it is not reliable! Several statistical, or psychometric, tests allow us to assess a tool’s reliability and validity (cfr. textbooks written on this topic). For instance, validation statistics of the tool may involve content validity, face validity, criterion validity, concurrent validity or predictive validity. *Step 10* is about verifying the stability, or robustness, of the aforementioned steps. It is the step in which you assess the significance, inference, and confidence (i.e., minimal measurement error) of your tool, using the sample(s) for which it was designed. *Step 11* involves standardization and norm development, allowing large-scale usage of your tool.

This review aims to conclude the trends associated with these questionnaires, and reinforce the importance of certain stages of tool development and highlight the direction of research that would be ideal to follow.

## Materials and Methods

**Figure 1:**
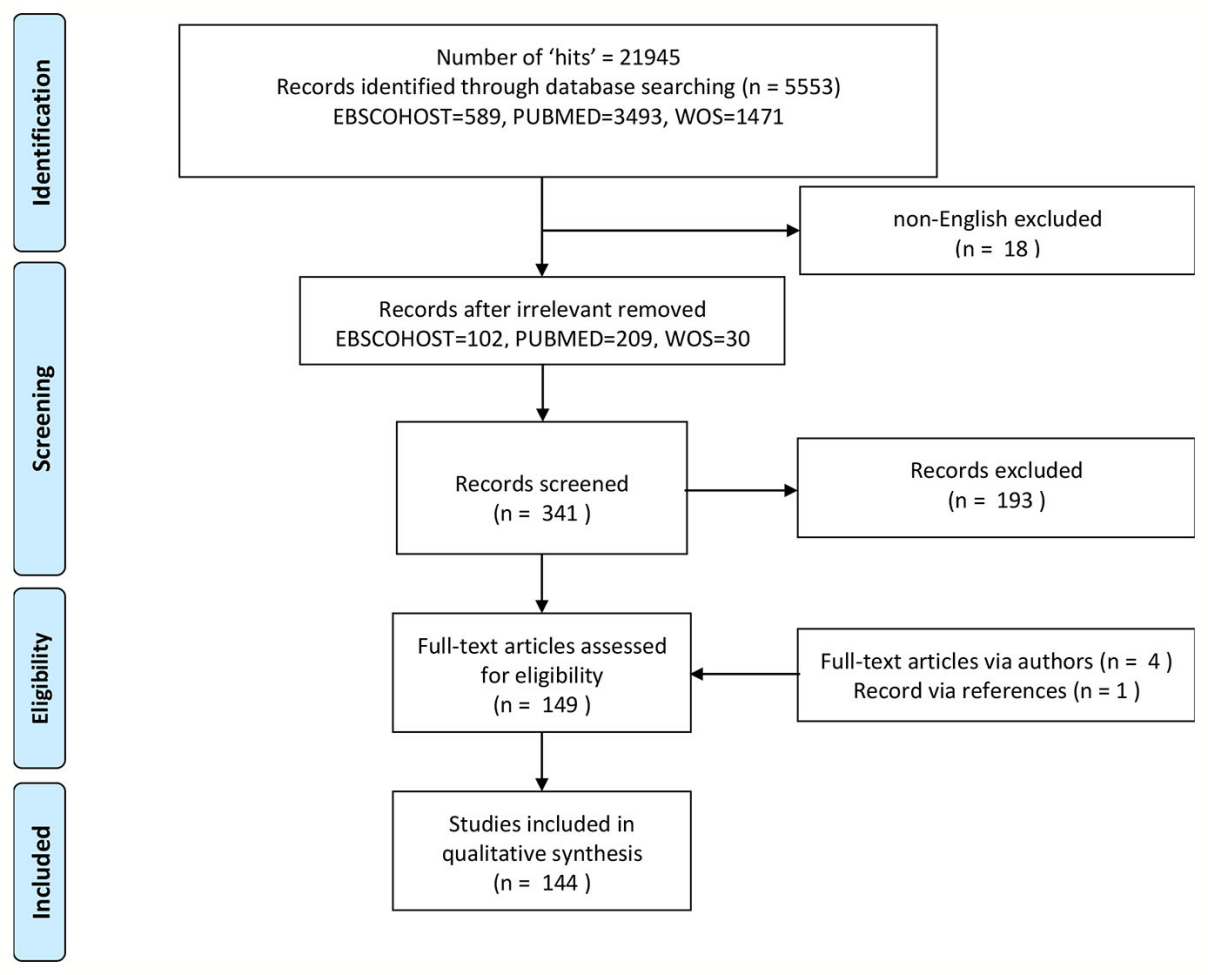
Flowchart of studies included.

To achieve consistency and retrieve relevant studies to the Spruyt (2, 3) review, the search terms(*) and databases were mirrored; *“Sleep” AND (“infant” OR “child” OR “adolescent”) AND (“questionnaire,” “instrument,” “scale,” “checklist,” “assessment,” “log,” “diary,” “record,” “interview,” “test,” “measure”).* The databases included PubMed, Web of Science (WOS), and EBSCOHOST (per PRISMA guidelines). Additional limitations to the search criteria were applied for date and age range of the respective study populations. Database-wide searches were conducted between 18^th^ of April 2010 (Spruyt, 2011 publication date of search) and 1^st^ of January 2020. Age categories listed in PubMed filters between 0 and 18 years were also applied to restrict the search to pediatric populations alone. Contrastingly, language criteria were not specified but post hoc constrained to English. Papers in other languages could not be evaluated by one of the authors, in case a consensus on the psychometric evaluation was needed. The search for relevant studies extended to authors in listserver groups PedSleep2.0 and the International Pediatric Sleep Association (IPSA) in order to achieve maximal inclusion. The refinement of these study characteristics ensured that the systematic review would evaluate relevant studies in pediatric tool development, adaptation, and validation. Final search count was sizeable (refer to **Figure 1**).

Full-text access was achieved through the literary database “Library Genesis” or author contact if necessary (see Acknowledgments). All flagged citations were then manually screened for relevant keywords in their respective titles, abstracts and methods to further refine studies relevant to the systematic review—these being 11 psychometric steps (2, 3) and 7 sleep categories (sleep quantity, sleep quality, sleep regularity, sleep hygiene, sleep ecology, and sleep treatment) (4). Consequently, independent studies were highlighted and screened, and each study’s descriptive variables were extracted and collated. Any absence of indispensable information regarding the tools use was addressed through contact of authors.

### Statistical Analysis

A total of 11 steps (2) and 7 sleep categories (4) were extracted and were statistically analyzed for frequency and descriptive assessment (refer to **Tables 1** and **2**). Any variables unmentioned or neglected were described as “empty,” and tabulated as such in the forthcoming interpretations. Continuous variables will be described as mean values (± standard deviation) and categorical variables will be shown as absolute and relative values. Statistical analyses were performed with Statistica version 13 (StatSoft, Inc. (2009), STATISTICA, Tulsa, OK).

**Table 1:** Basic information of studies evaluated.

Tool acronym	First author	Year	Place of origin	Sample size	Age (years)	Number of questions	Scale	Respondent	Timeframe	Reference has questionnaire	Steps fulfilled
**AIS** (5)	Chung	2011	Hong Kong, China	1,516	12–19	8	three-point Likert	self	in the last month	no	1,2,4,5,6,7,8,9
*setting* : three schools with different levels of academic achievement											
**ASHS** (6)	Storfer-Isser	2013	Boston, USA	514	16–19	32	six-point ordinal	self	in the past month	no	1,2,6,7,8,9,10
*setting* : Cleveland Children's Sleep and Health Study, a longitudinal, community-based urban cohort study											
**ASHS** (7)	de Bruin	2014	Amsterdam, Netherlands	186 normal and 112 insomnia	12–19	28	six-point rating	self	in the past month	yes	1,2,8,9
*setting* : a community sample of adolescents and a sample of adolescents with insomnia (registered through a website)											
**ASHS** (8)	Chehri	2017	Basel, Switzerland	1,013	12–19	24	six-point rating	self	in the past month	no	1,2,4,6,7,8,9,10
*setting* : classroom – individual											
**ASHS** (9)	Lin	2018	Qazvin, Iran	389	14–18	24	six-point rating	self	in the past month	no	1,2,4,5,6,7,8,9,10
*setting* : classroom – individual											
**ASQ** (10)	Arroll	2011	Auckland, New Zealand	36	>15	30	mixed	self	mixed	yes	1,2,3,4,5,6,9
*setting* : primary care patients											
**ASWS** (11)	Sufrinko	2015	north Carolina, USA	467	12–18	10		self		no	1,2,6,7,8,9,10
*setting* : classroom – individual											
**ASWS** (12)	Essner	2015	Seattle, USA	491	12–18	28	six-point Likert	self	previous month	no	1,2,7,8,9
*setting* : data were pooled from five research studies with heterogeneous samples of adolescents with nondisease-related chronic pain, sickle cell disease, traumatic brain injury, or depressive disorders, as well as adolescents who were otherwise healthy, from three sites in the Northwest and Midwestern United States.											
**BEARS** (13)	Bastida-Pozuelo	2016	Murcia, Spain	60	2–16	7	yes/no	parent		no	1,2,4,6,9
*setting* : first time visit at National Spanish Health Service's mental healthcare centre											
**BEDS** (14)	Esbensen	2017	Ohio, USA	30	6–17	28	five-point Likert	parent	in last 6 months	no	1,2,6,8,9
*setting* : take-home questionnaires and sleep diary											
**BISQ** (15)	Casanello	2018	Barcelona, Spain	87	3–30 months	14	mixed	parent		yes	1,2,4,5,6,8,9
*setting* : clinic based (self-report and follow-up interview)											
**BRIAN-K** (16)	Berny	2018	Porto Alegre, RS, Brazil	373	7–8	17	three-point Likert	parent	in the last 15 days	yes	1,2,3,4,5,6,7,8,9
*setting* : classroom – individual											
**CAS-15** (17)	Goldstein	2012	New York, USA	100	2–12	15	mixed	clinician		yes	all steps except 10
*setting* : children referred to the pediatric otolaryngology outpatient offices for evaluation of snoring and suspected sleep disordered breathing											
**CBCL** (18)	Becker	2015	Cincinnati, OH, USA	383	6–18	7 sleep items	three-point Likert	parent/self		no	1,2,6,8,9
*setting* : referred patients to tertiary-care pediatric hospital											
**CCTQ** (19)	Dursun	2015	Erzurum, Turkey	101	9–18	27	mixed	parent	on work and free days	no	1,2,6,8,9
*setting* : sample from clinical (outpatient psychiatry) and community settings											
**CCTQ** (20)	Ishihara	2014	Tokyo, Japan	346	3–6	27	mixed	parent	on work and free days	no	1,2,6,8,9
*setting* : mailed to parents *via* kindergartens											
**CCTQ** (21)	Yeung	2019	Hong Kong, China	555	7–11	27	mixed	parent		no	1,2,3,4,5,6,8,9
*setting :* five primary schools in the Hong Kong SAR											
**CRSP** (22)	Cordts	2016	Kansas, USA	155	9.82	62		self		no	1,2,6,7,9,10
*setting* : take-home questionnaire/classroom group											
**CRSP** (23)	Meltzer	2013	Denver, Colorado, USA	456	8–12	60	mixed	self	mixed	yes	1,2,4,8,9,10
*setting:* primary care pediatricians' offices, an outpatient pediatric sleep clinic, community flyers and advertisements, two independent Australian schools, two different pediatric sleep laboratories, and outpatient clinics or inpatient units of a children's hospital for oncology patients											
**CRSP** (24)	Meltzer	2014	Denver, Colorado, USA	570	13–18	76	mixed	self	mixed	no	1,2,4,7,8,9,10
*setting*: from several studies: pediatric sleep clinics at two separate children's hospitals, outpatient clinics and inpatient units of a children's hospital for oncology patients, two independent Australian schools, an Internet based sample of adolescents, including those with asthma (categorized in clinic group) and those without asthma (categorized in community group)											
**CRSP** (25)	Steur	2019	Amsterdam, Netherlands	n= 619 general n=34 clinic	7–12	26 (total score on 23)	three-point	self	one week	no (English items listed)	1,4,7,8,9,10,11
*setting* : online data collection in cooperation with the Taylor Nelson Sofres Netherlands Institute for Public Opinion, an outpatient sleep clinic											
**CRSP-S** (26)	Meltzer	2012	Denver, Colorado, USA	388	8–12	5	5-point rating	self		no	1,2,6,7,8,9,10
*setting* : primary care pediatrician's offices: the Sleep Clinic at the Children's Hospital of Philadelphia (CHOP), through community flyers and advertisements in the Delaware Valley, through two independent schools in Adelaide, South Australia, while waiting for an overnight polysomnography at CHOP or the Children's Hospital of Alabama, or during outpatient clinic visits or on the inpatient unit at St. Jude Children's Research Hospital											
**CSAQ** (27)	Chuang	2016	Taichung, Taiwan	362	8–9	44	four-point Likert	parent		no	all steps except 11
*setting* : elementary school											
**CSHQ** (28)	Markovich	2015	Halifax, Canada	30	6–12	45 (33 scored question)	three-point Likert	parent	in the previous week	no	1,2,8,9
*setting* : data were collected from two larger studies											
**CSHQ** (29)	Dias	2018	Braga, Portugal	299	2 weeks–12 months	48	four-point Likert	parent	mixed	yes	1,2,4,5,6,7,8,9
*setting* : women were contacted at the third trimester of pregnancy; send by email											
**CSHQ** (30)	Ren	2013	Beijing, China	912	6–12	33	three-point Likert	parent		no	1,2,6,7
*setting* : Parent meeting at primary and elementary students in Shenzhen											
**CSHQ** (31)	Liu	2014	Chengdu, China	3,324	3–6	33	three-point Likert	parent	a typical week	no	1,2,6,7,8,9,10
*setting* : 21 mainland Chinese cities; take-home questionnaire											
**CSHQ** (32)	Tan	2018	Shanghai, China	171	4–5	33	three-point and four-point Likert	parent		no	1,2,6,7,8,9,10
*setting* : distributed at the schools; take-home questionnaire											
**CSHQ** (33)	Waumans	2010	Amsterdam Netherlands	1,502	5–12	33	four-point Likert	parent		no	1,2,4,5,6,7,8,10
*setting* : primary schools and daycare centers											
**CSHQ** (34)	Steur	2017	Amsterdam Netherlands	201	2–3	33	three-point Likert	parent	1-week	no	1,2,4,6,7,8,10,11
*setting* : online questionnaire *via* a Dutch market research agency											
**CSHQ** (35)	Mavroudi	2018	Thessaloniki, Greece	112	6–14	45	four-point Likert	parent	a “common” recent week	no	1,2,8,9
*setting* : patients were ascertained sensitive to a variety of aeroallergens											
**CSHQ** (36)	Johnson	2016	Florida USA	310 (177+34+99)	2–10	33	a 1–3 rating + yes/no	parent		no	1,2,6,7,8
*setting* : enrolled from three study sites : 24-week, multisite randomized controlled trial of parent training (PT) versus parent education; an 8-week randomized trial of a PT program; Autism Speaks Autism Treatment Network											
**CSHQ** (37)	Sneddon	2013	Vancouver, BC, Canada	105	2–5	33	three-point Likert	mother		no	1,2,6,7,8,9
*setting* : early intervention programs, outpatient mental health clinics; general community											
**CSHQ** (short) (38)	Masakazu	2017	Tokyo, Japan	178; 432; 330	6–12	19	three-point rating	parent	a typical recent week	no	1,2,3,4,5,6,8,9,10
*setting* : different collection times/settings: elementary school; pediatric psychiatric hospital; community											
**CSHQ** (39)	Schlarb	2010	Tübingen, Germany	298;45	4–10	48	three-point + yes/no	parent		no	1,2,4,6,7,8,9
*setting* : community sample *via* schools, clinical sample											
**CSHQ** (40)	Silva	2014	Lisbon, Portugal	315	2–10	33	three-point rating	parent	a recent more typical week	no	1,2,4,5,6,7,8,9
*setting* : community sample											
**CSHQ** (41)	Lucas-de la Cruz	2016	Cuenca, Spain	286	4–7	33	three-point rating	parent		no	1,2,4,6,7,8,9
*setting* : cross-over cluster randomized trial from 21 schools											
**CSHQ** (42)	Fallahzadeh	2015	Kashan, Iran	300	5–10	33	three-point rating	parent		no	1,2,4,5,6,7,8,9
*setting* : public and private schools											
**CSHQ** (43)	Loureiro	2013	Lisbon, Portugal	574	7–12	26	three-point Likert	parent		no	1,2,4,5,6,8,9
*setting* : community and clinical samples											
**CSHQ** (short) (44)	Bonuck	2017	Boston, Masacheusettes	151;218	4–10; 24–66 months	23		parent		no	1,2,6,9
*setting* : clinic sample data (two datatest were reused for this study: Owens (1997/8) and Goodlin-Jones (2003-5), respectively)											
**CSHQ** (14)	Esbensen	2017	Cincinnati, OH, USA	30	6–17	33	three-point Likert	parent		no	1,2,6,8,9
*setting*: community-based study in children with Down syndrome											
**CSM** (45)	Jankowski	2015	Warsaw, Poland	952	13–46	13	mixed	self		yes	1,2,4,6,8,9
*setting* : residents from Warsaw and Mielec districts											
**CSRQ** (46)	Dewald	2012	Amsterdam Netherlands	166; 236	12.2–16.5; 13.3–18.9	20	ordinal response categories ranging from 1 to 3	self	previous 2 weeks	no	1,2,4,6,7,8,10
*setting* : five high schools in and around Amsterdam and from five high schools in Adelaide and Outer Adelaide											
**CSRQ** (47)	Dewald-Kaufmann	2018	Amsterdam Netherlands	298		20	ordinal response categories ranging from 1 to 3	self	previous 2 weeks	no	1,2,9,11
*setting :* participants were recruited from high schools around Amsterdam; referred to the Centre for Sleep–Wake Disorders and Chronobiology of Hospital Gelderse Vallei in Ede, the Netherlands; adolescents who received cognitive behavioural therapy for their sleep onset and maintenance problems (see de Bruin et al)											
**CSWS** (48)	LeBourgeois	2016	Boulder, CO, USA	161; 485; 751; 55;85	2–8 (different across studies)	25 (different across studies)	four-point (different across studies)	parent		no	all steps except 11
*setting* : 5 studies with independent samples (different across studies)											
**DBAS** (49)	Lang	2017	Basel, Switzerland	864	17.9	16	10-point Likert	self		no	1,2,4,6,7,8,9,10
*setting* : students in vocational education and training; in a classroom setting											
**DBAS** (50)	Blunden	2012	Queensland Australia	134	11–14	10	mixed	self		no	1,2,3,4,5,6,7,8,9
*setting* : From sleep education intervention											
**ESS** (51)	Krishnamoorthy	2019	Puducherry, India	789	10–19	8	four-point Likert	self		no	all steps
*setting* : villages of rural Puducherry, a union territory in South India											
**ESS** (52)	Crabtree	2019	Memphis, Tennessee	66	6–20	8	four-point Likert	self	in various everyday situations	no	1,2,8,9,11
*setting :* children and young adults (ages 6 to 20 years) were assessed by the M-ESS after surgical resection, if performed, and before proton therapy											
**ESS-CHAD** (53)	Janssen	2017	Victoria, Australia	297	12–18	8	four-point Likert	self	thinking of the last two weeks	no	1,2,6,7,8,9,10
*setting* : Part of a broader research project; schools in regional Victoria (qualtrics survey)											
**FoSI** (54)	Brown	2019	Washington, DC, USA	147	14–18	11	five-point Likert	self	last month	no	1,2,6,7,8,9,10
*setting :* two school-based health centers in the Washington Metropolitan Area											
**I SLEEPY** (55)	Kadmon	2014	Ontairo, Canada	150	3–18	8	yes/no	parent/self		yes	1,2,4,5,6,9
*setting* : referred for evaluation at a pediatric sleep clinic											
**IF SLEEPY** (55)	Kadmon	2014	Ontairo, Canada	150	3–18	8	yes/no	parent/self		yes	1,2,4,5,6,9
*setting* : referred for evaluation at a pediatric sleep clinic											
**I'M SLEEPY** (55)	Kadmon	2014	Ontairo, Canada	150	3–18	8	yes/no	parent/self		yes	1,2,4,5,6,9
*setting* : referred for evaluation at a pediatric sleep clinic											
**ISI** (5)	Chung	2011	Hong Kong, China	1,516	12–19	8	five-point Likert	self	in last 2 weeks	no	1,2,4,5,6,7,8,9
*setting* : three schools with different levels of academic achievement											
**ISI** (56)	Kanstrup	2014	Solna, Sweden	154	10–18	5	five-point rating	self	past 2 weeks	no	1,2,4,6,8,9
*setting* : patients with chronic pain referred to a tertiary pain clinic upon first visit											
**ISI** (57)	Gerber	2016	Basel, Switzerland	1,475 adolescents, 862 university students and 533 adults	11–16	7	eight-point Likert	self		yes	1,2,4,6,7,8,9,10
*setting* : 3 cross-sectional studies; *via* schools											
**JSQ** (58)	Kuwada	2018	Osaka, Japan	4,369; 100	6–12	38	mixed (6 point intensity rating)	parent		no	1,2,7,8,9,10,11
*setting* : 17 elementary schools; 2 pediatric sleep clinic											
**JSQ** (preschool) (59)	Shimizu	2014	Osaka, Japan	2,998;102	2–6	39	six-point Likert	parent		no	1,2,4,6,7,8,9,11
*setting* : private kindergarten, nursery school, and recipients of regular physical examinations at the age of 3 years; two pediatric sleep clinics											
**LSTCHQ** (60)	Garmy	2012	Lund, Sweden	116 child respondents; 44 parent respondents	6–13	11	mixed	parent/self		yes	1,2,4,5,8,9
*setting* : school-based distriution											
**MCTQ** (61)	Roenneberg	2003	Basel, Switzerland	500 (142 being <21years)	6–18	~9*	seven-point rating; mixed	self	free/work days	yes	1,2,5,6
*setting* : distributed in Germany and Switzerland in high schools, universities, and the general population. This paper was added because of its relevance despite being outside the timeframe of the current review											
**MEQ** (62)	Cavallera	2015	Milan, Italy	292	11–15	17		self		no	1,2,4,5,7,8,9
*setting* : convenience school-based samples											
**(r)MEQ** (63)	Danielsson	2019	Uppsala, Sweden	671	16–26	5		self		no	1,2,6,7,8,9
*setting :* selected randomly from the Swedish Population Register											
**aMEQ** (64)	Rodrigues	2016	Aveiro district, Portugal	300	12–14	19	mixed	self		no	1,2,4,5,6,8,9,11
*setting:* 80% public and 20% private schools from the district of Aveiro											
**aMEQ-R** (65)	Rodrigues	2019	Aveiro district, Portugal	n1=300 (same 2016) n2= 217	12–14	10	mixed	self		no	1,2,4,5,6,8,9,11
*setting:* several schools of the Aveiro district											
**MESC** (66)	Diaz-Morales	2015	Madrid, Spain	5,387	10–16			self		no	1,2,4,6,7,8,9,10
*setting*: public high schools in Madrid and the surrounding area											
**MESSi** (67)	Demirhan	2019	Sakarya, Turkey	1,076	14–47	15	five-point Likert	self		yes	1,4,5,7,8,9,10
*setting:* high school and university students											
**MESSi** (68)	Weidenauer	2019	Tuebingen, Germany	215	11–17	15	five-point Likert	self		yes	1,6,8,9,10
*setting:* three different gymnasia (highest stratification level of school teaching) in SW Germany, Baden-Wuerttemberg											
**My Sleep and I** (69)	Rebelo-Pinto	2014	Lisbon, Portugal	654	10–15	27	five-point Likert	self		no	1,2,3,4,7,8,9,10
*setting*: schools in Portugal part of project Sleep More to Read Better											
**My children's sleep'** (69)	Rebelo-Pinto	2014	Lisbon, Portugal	612	21–68	27	five-point Likert	parent		no	1,2,3,4,7,8,9,10
*setting*: schools in Portugal part of project Sleep More to Read Better											
**NARQoL-21** (70)	Chaplin	2017	Gothenburg, Sweden	158	8–13; 15–17	21	five-point Likert	self		no	all steps
*setting* : patient and control group											
**NSD** (71)	Yoshihara	2011	Tochigi, Japan	40	6 months–6 years	2		parent	diary	yes	1,2,3,4,5,6
*setting* : take home diary											
**NSS** (72)	Ouyang	2019	Beijing, China	n=53 pediatric n= 69 adult	>8 years	15				no	1, 2, 7, 8, 9
*setting* : sleep lab											
**OSA Screening Questionnaire** (73)	Sanders	2015	Southampton, UK		infancy to 6 years	33		parent	over a week	yes	1,2,3,4,5,6,9
*setting* : *via* a local Down syndrome parent support group											
**OSA-18 Questionnaire** (74)	Huang	2015	Hsinchu, Taiwan	163	6–12	18	seven-point ordinal	parent	past 4 weeks	yes (English)	1,2,4,7,8,9,10
*setting* : *via* schools											
**OSA-18 Questionnaire** (75)	Kang	2014	Taipei, Taiwan	109	2–18	18	seven-point ordinal	parent		yes	1,2,4,6,8,9
*setting* : recruited from the respiratory, pediatric, psychiatric, and otolaryngologic clinics											
**OSA-18 Questionnaire** (76)	Bannink	2011	Rotterdam, Netherlands	119 patients; 162 (child);459 parent	2–18	18; OSA-12 in children, OSA-18 in parents	seven-point ordinal	parent/self		yes	1,2,4,6,8,9
*setting* : patients with syndromic craniosynostosis; convenience sample of parents											
**OSA-18 Questionnaire** (77)	Mousailidis	2014	Athens, Greece	141	3–18	18	seven-point ordinal	parent		yes	1,2,4,6,8,9
*setting* : children who were referred for overnight polysomnography at the Sleep Disorders Laboratory											
**OSA-18 Questionnaire** (78)	Fernandes	2013	Guimarães, Portugal	51	2–12	18	seven-point ordinal	parent	past 4 weeks	yes (English)	1,2,4,5,6,8,9
*setting* : sleep clinic											
**OSA-18 Questionnaire** (79)	Chiner	2016	Alicante, Spain	60	2–14	18	seven-point ordinal	parent	4 weeks	yes	1,2,4,6,7,8,9
*setting* : children with suspected apnea-hypopnea syndrome were studied with polysomnography											
**OSA-5 Questionnaire** (short) (80)	Soh	2018	Melbourne, Australia	366 and 123	2–17.9	5	four-point Likert	parent	past 4 weeks	yes	all steps except 11
*setting*: Melbourne Children's Sleep Centre for polysomnography											
**OSD-6 QoL Questionnaire** (81)	Lachanas	2014	Larissa, Greece	91	3–15	6	seven-point ordinal	parent		yes (Greek and English)	1,2,4,5,6,8,9
*setting* : children undergoing polysomnography											
**oSDB and AT** (82)	Links	2017	Baltimore, USA	32		39	three-point rating	parent		yes	1,2,4,6,8,9
*setting* : online Questionnaire											
**OSPQ** (83)	Biggs	2012	Adelaide, Australia	1,904	5–10	26	four-point Likert	parent	last typical school week	no	1,2,4,5,6,7,8,10,11
*setting* : *via* 32 elementary schools in Adelaide											
**PADSS** (84)	Arnulf	2014	Paris, France	73; 98	>15	17		self		no	1,2,3,4,5,6,7,8,9
*setting* : patients with sleepwalking or sleep terror referred to the sleep disorder unit; controls											
**PDSS** (85)	Felden	2015	Curitiba, Brazil	90	10–17	8	five-point Likert	self		yes	1,2,4,5,8,9
*setting* : two private schools											
**PDSS** (86)	Komada	2016	Tokyo, Japan	492	11–16	8		self		no	1,2,4,5,6,7,8,9
*setting* : one elementary school, one junior high school and one high school, located in suburbs of Japan											
**PDSS** (87)	Bektas	2015	Izmir, Turkey	522	5–11	8	four-point Likert	self		no	1,2,4,5,6,7,8,9,10
*setting* : students were in grade 5-11											
**PDSS** (88)	Ferrari Junior	2018	Florianópolis, SC, Brazil	773	14–19	8	five-point Likert	self		no	1,7,8,9,10
*setting :* state schools of Paranaguá, Paraná											
**PDSS** (89)	Randler	2019	Petrozavodsk, Russia	n1= 285 n2= 267 n3= 204	7–12	8	five-point Likert	self		yes	1,2,4,5,6,7,8,9,10
*setting :* Schools from six different settlements located in North-Western Russia (Murmansk region) participated in the study during our framework project "Sleep Health in Russian Arctic"											
**Pediatric Sleep CGIs** (90)	Malow	2016	Nashville, USA	20	5.3	14	seven-point rating	parent		yes (link)	1,2,4,5,6,9
*setting* : participants in a 12-week randomized trial of iron supplementation in children with autism spectrum disorders											
**PedsQL (fatigue scale)** (91)	Al-Gamal	2017	Amman, Jordan	70	5–18	18	three- and five-point Likert	self		no	1,2,4,5,6,8,9
*setting* : oncology outpatient clinic											
**PedsQL (fatigue scale)** (92)	Qimeng	2016	Guangzhou, China	125	2–4	18	five-point Likert	parent		no	1,2,4,5,6,7,8,9
*setting* : diagnosed to have acute leukemia for 1 month at the least											
**PedsQL(fatigue scale)** (93)	Nascimento	2014	São Paolo, Brazil	216; 42 children (8–12 years), 68 teenagers (13–18 years), and 106 caregivers (parents or guardians)	8–18	18	five-point Likert	parent/self		no	1,2,4,6,7,8,9,10
*setting* : oncology inpatient and outpatient pediatric clinics											
**PISI** (94)	Byars	2017	Cincinnati, OH, USA	462	4–10	6	six-point Likert	parent		yes	1,2,4,6,7,8,9,10
*setting* : behavioral sleep medicine evaluation clinic											
**PNSSS** (95)	Whiteside-Mansell	2017	Little Rock, Arkansas, USA	72	1 week to 28 weeks	14	four-point scale	professional		no	1,2,8
*setting* : a naturalistic study of participants enrolled in two home visitation support programs											
**PosaST** (96)	Pires	2018	Porte Alegre, Brazil	60	3–9	6	five-point rating	self		yes	1,2,4,5,8,9
*setting* : children undergoing polysomnography											
**PPPS** (97)	Finimundi	2012	Porto Alegre, Brasil	144	10–17	mixed	five-point rating	self		no	1,2,9
*setting :* adolescent students attending elementary school in two public schools in the state of Rio Grande do Sul (municipalities of Esteio and Farroupilha – great Porto Alegre, and Serra Gaúcha											
**P-RLS-SS** (98)	Arbuckle	2010	Cheshire, United Kingdom	cognitive debriefing interviews with 21 of the same children/adolescents and 15 of their parents	6–17	26 morning and 28 evening items	Wong and Baker pain faces scale	parent/self		no	1,2,4,5,6
*setting* : four pediatric sleep disorders specialists											
**PROMIS** (99)	van Kooten	2016	Amsterdam, Netherlands	6 experts, 24 adolescents and 7 parents	12–18	27 (PROMIS-SD), 16 (PROMIS-SRI)	through Computerized AdaPOINTive Testing	self/parent/expert		no	1,2,9
*setting* : distributed to the adolescents in the classroom											
**PROMIS** (100)	van Kooten	2018	Amsterdam, Netherlands	1,046	11–19	27 (PROMIS- Sleep Disturbance), 16 (PROMIS- Sleep-Related Impairment)		Self		no	1,2,6,7,9,10
*setting* : online; schools from all educational levels and from different regions of the Netherlands											
**PROMIS** (101)	Forrest	2018	Philadelphia, PA, USA	1,104 children (8–17 years old) and 1,477 parents of children 5–17 years old	5–17	43; the final item banks included 15 items for Sleep Disturbance and 13 for Sleep-Related Impairment	frequency-based (1: never, 2: almost never, 3: sometimes, 4: almost always, 5: always)	self/parent	7-day	yes	1,2,6,7,8,9,10
*setting* : a convenience sample of children and parents recruited from a pediatric sleep clinic											
**PROMIS** (102)	Bevans	2019	Philadelphia, PA, USA	8 expert sleep clinician-researchers, 64 children ages 8–17 years, and 54 parents of children ages 5–17 years	children ages 8–17 and parents of children ages 5–17.	The final item pool contains 43 child-report items and 49 parent-report items	five-point Likert	Self/Parent	In the past 7 days	yes	1,2,3,4,5,6,9
*setting :* A preliminary child sleep health conceptual framework was generated based on the two PROMIS Adult Sleep Health item banks. Thereafter, the framework was refined based on expert and child and parent interviews											
**PSIS** (103)	Smith	2014	Texas, USA	155	3–5	12	five-point Likert	parent		no	1,2,6,8,9
*setting* : identified using a commercial mailing list and print advertisements distributed throughout local schools, daycares, community centers, and health care providers											
**PSQ** (104)	Ishman	2016	Ohio, USA	45	16.7	22	yes/no/don't know	parent		no	1,2,6,8
*setting* : teen-longitudinal assessment of bariatric surgery (Teen-LABS) participants at high-risk for obstructive sleep apnea											
**PSQ** (105)	Yüksel	2011	Manisa, Turkey	111	2–18	22	yes/no and I don't know	parent		no	1,2,4,5,6,8,9
*setting* : pediatric allergy and pulmonology outpatient department											
**PSQ** (106)	Bertran	2015	Santiago, Chile	83	0–15	22	yes/no/don't know	parent		no	1,2,6,7
*setting*: habitually snoring children referred for polysomnography											
**PSQ** (107)	Hasniah	2012	Kuala Lumpur, Malaysia	192;554	6–10	22	"yes=1," "No=0," and "Don't know=Missing"	parent		no	1,2,4,5,6,8,9
*setting* : part of the national epidemiological study of the prevalence of sleep-disordered breathing in Malaysian school children											
**PSQ** (108)	Chan	2012	Hong Kong, China	102	2–18	22	yes/no/don't know	parent		no	1,2,9,11
*setting* : underwent overnight sleep polysomnography studies for suspected OSA in the sleep laboratory											
**PSQ** (109)	Ehsan	2017	Cincinatti, USA	160	2–18	22	yes/no/don't know	parent		no	1,2,6,9
*setting* : using an existing clinical database encompassing all children referred to the Cincinnati Children's Hospital Sleep Center for polysomnography											
**PSQ** (110)	Li	2018	Beijing, China	9,198	3.0–14.4	22	yes/no/don't know	parent		no	1,2,6,7,8,9
*setting* : 11 kindergartens, 7 primary schools and 8 middle schools from 7 districts of Beijing, China											
**PSQ** (111)	Longlalerng	2018	Chiang Mai, Thailand	62	7–18	22	yes/no/don't know	parent		no	1,2,4,5,8,9
*setting* : clinic based retrieval classified as overweight or obese according to the International Obesity Task Force and diagnosed with obstructive sleep apnea											
**PSQ** (112)	Raman	2016	Ohio, USA	636	4–25.5	36		parent		yes	1,2,4
*setting* : patients scheduled for a sleep study											
**PSQ** (113)	Certal	2015	Porto, Portugal	180	4–12	22	yes/no	self		yes	1,2,4,5,6,8,9
*setting* : *via* schools north Portugal											
**PSQ** (114)	Jordan	2019	Paris, France	201	2–17	22	"yes," "no" or "don't know,"	parent		yes	1,2,4,5,6,7,8,9,10
*setting* : admitted to the Odontology Center of the Rothschild Hospital (Assistance Publique e Hopitaux de Paris)											
**PSQI** (115)	Passos	2017	Pernambuco, Brazil	309	10–19	19	0–3 rating	self		no	1,2,4,5,6,7,8,9,10
*setting* : subjects who engaged in amateur sports practice											
**PSQI** (116)	Raniti	2018	Melbourne, Australia	889	12.08–18.92	18	four-point Likert scale	self	1 month	no	1,7,8,9,10
*setting :* 14 Australian secondary schools											
**RLS** (117)	Schomöller	2019	Potsdam, Germany	33 (11 RLS)	6–12 and 13–18	12	mixed	self/parent		yes	1,2,3,4,6,8,9
*setting :* with the support of medical somnologists, who recruited pediatric patients from their practice or sleep laboratories, newsletter announcements in the Restless Legs Association journal, and *via* local selfhelp groups.											
**SDIS** (118)	Graef	2019	Cincinnati, Ohio	392	2.5–18.99	SDIS-C, 41 items, 2.5–10 years; SDIS-A, 46 items, 11–18 years	seven-point Likert scale	parent		no	1,9
*setting :* Youth with insomnia, of whom 392 underwent clinically indicated diagnostic PSG within ± 6 months of SDIS screening											
**SDPC** (119)	Daniel	2016	Philadelphia, USA	20;6	3–12	41	0–4 rating	parent	Interview modelling	no	1,2,4,6,9
*setting* : parents of children with acute lymphoblastic leukemia and medical providers											
**SDSC** (120)	Huang	2014	Guangzhou, China	3,525	5–16	26	five-point scale	parent	six months	no	1,2,4,5,6,7,8,9,10,11
*setting* : selected from five primary schools in Shenyang											
**SDSC** (121)	Putois	2017	Sierre, Switzerland	447	4–16	25	five-point scale	parent	six months	yes	1,2,4,5,6,7,8,9,10,11
*setting*: schools; pediatric sleep clinic											
**SDSC** (122)	Saffari	2014	Isfahan, Iran	100	6–15	26	five-point scale	parent	six months	no	1,2,4,5,6,8,9
*setting*: primary and secondary schools in Isfahan City, Iran											
**SDSC** (14)	Esbensen	2017	Cincinnati, OH, USA	30	6–17	26	five-point scale	parent	6 months	no	1,2,6,8,9
*setting*: part of a larger community-based study down syndrome sample											
**SDSC** (123)	Cordts	2019	Portland, OR, USA	69	3–17	26	five-point Likert	parent	6 months	no	1,6,8,9
*setting*: longitudinal pediatric neurocritical care programs at two tertiary academic medical centers within 3 months of hospital discharge											
**SDSC** (124)	Mancini	2019	Western Australia, Australia	307	4–17	26	five-point Likert	parent	6 months	no	1,2,10
setting: recruited *via* the Complex Attention and Hyperactivity Disorders Service (CAHDS), in Perth, Western Australia											
SDSC* (125)	Moo-Estrella	2018	Yucatán, Mexico	838	8–13	25	number of days : 0 = 0 days, 1 = 1–2 days, 2 = 3–4 days, 3 = 5–6 days, and 4 = 7 days.	self	during the last week	no	1,2,3,4,5,6,7,8,9
*setting* : between the third and sixth grades of elementary school, recruited by convenience sampling											
**SHI** (126)	Ozdemir	2015	Konya, Turkey	106 patients with major depression; 200 volunteers recruited from community sample	16–60	13	Always, Frequently, Sometimes, Rarely, Never	self		no	1,2,6,7,8,9,10
*setting* : university based retrieval											
**SHIP** (127)	Rabner	2017	Boston, USA	1,078	7–17	15	three-point Likert	parent/self		no	1,2,6,8,9
*setting*: parents and children each completed questionnaires individually within 1 week prior to the child's multidisciplinary headache clinic evaluation											
**Sleep Bruxism** (128)	Restrepo	2017	Medellın, Colombia	37	8–12	1	yes/no	parent	5-day diary	yes (English)	1,2,4
*setting* : recruited from the clinics at Universidad CES											
**SNAKE** (129)	Blankenburg	2013	Datteln, Germany	224	<10	54	1–4 rating (mixed)	parent		yes (English)	all steps
*setting* : children with severe psychomotor impairment; questionnaire-based, multicenter, cross-sectional survey											
**SQI** (5)	Chung	2011	Hong Kong, China		12–19	8	three-point Likert	self	In past 3 months	no	1,2,4,5,6,7,8,9,10
*setting*: three schools with different levels of academic achievement											
**SQ–SP** (130)	Maas	2011	Maastricht, Netherlands	345	1–66	45	seven-point Likert	parent	last three months	yes	1,2,6,7,8,9,10,
*setting*: individuals who consulted the sleep clinic for individuals with ID; individuals from a control group who attended a special day care center, special school or adult activity center for individuals with ID; participants of two published studies Maas et al., 2008, 2009); individuals who consulted a psychiatric clinic for children and adolescents with ID											
**SQS-SVQ** (131)	Önder	2016	Sakarya, Turkey	1,198	11–15	15*		self		yes	1,2,4,7,8,9,10
*setting*: an instrument adaptation study with different groups											
**SRSQ** (132)	van Maanen	2014	AmsterdamNetherlands	951;166;236;144;66	14.7 (mean)	9	three-point ordinal	self	previous 2 weeks	no	1,2,6,8,9
*setting* : various samples from the general and clinical populations; online and paper and pencil											
**SSR** (133)	Orgilés	2013	Alicante, Spain	1,228	8–12	26	three-point	self		yes	1,2,4,6,7,8,9,10
*setting* : 9 urban and suburban schools; per 20 in group											
**SSR** (43)	Loureiro	2013	Lisbon, Portugal	306	7–12	26	three-point	self		no	1,2,4,5,6,8,9
*setting* : community and clinical samples											
**SSSQ** (134)	Yamakita	2014	Koshu, Japan	58	9–12	Please note your bedtime and wake time on both weekdays and weekends		self	log	no	1,2,8,9
*setting* : a typical elementary school in Koshu City											
**STBUR** (135)	Tait	2013	Michigan, USA	337	2–14	5	yes/no, and don't know	parent		yes	1,2,3,4,6,7
*setting* : parents of children scheduled for surgery											
**STQ** (136)	Tremaine	2010	Adelaide, Australia	65	11–16	18	time	self		no	1,2,9
*setting* : 3 different private (independent) schools in South Australia											
**The Children's Sleep Comic** (137)	Schwerdtle	2012	Landau, Germany	201	5–10	37	tick in applicable square	self		no (examples)	1,2,4,9
*setting* : three primary schools in Germany (group)											
**The Children's Sleep Comic** (138)	Schwerdtle	2015	Würzburg, Germany	176;393	5–11	20	tick in applicable square	parent/self		no (examples)	1,2,3,4,6,8,9,11
*setting* : three primary schools in Germany (group)											
**TuCASA** (139)	Leite	2015	São Paolo, Brazil	62	4–11	13		parent		yes	1,2,4,8,9
*setting* : sleep-disordered breathing diagnosed by polysomnography and controls											
**YSIS** (140)	Liu	2019	Shandong Province, China	11,626	15.0 ±1.5	8	five-point Likert	self	past month	yes	1,2,4,5,6,7,8,9,10,11
*setting* : Shandong Adolescent Behavior and Health Cohort, five middle and three high schools in three counties of Shandong Province, China											

Steps: 1: purpose; 2: research question; 3: response format; 4: generate items; 5: pilot; 6: item-analysis, nonresponse; 7: structure; 8 reliability; 9: validity; 10: confirmatory analyses; 11: standardize and develop norms

## Results

### Studies Included

As described by **Figure 1**, the total number of studies generated from the database search was sizeable, at n=341. Key emphasis of a pediatric diagnostic tools’ use, development or validation deemed it eligible for review, as well as the general translation and consequent adaptation of any pediatric questionnaire, survey, log, diary, etc. The titles and abstracts of each report were screened accordingly, resulting in the omission of 193 articles and final inclusion of 144 articles. Exported abstracts were then assigned their respective full-text. Complete text access was not available for 14, while retrieved from either the literature database “Library Genesis” or *via* author permission (n=4, see Acknowledgments), leaving 144 or 70 tools eligible for review based on the search conducted.

A more thorough examination of methodological processes was then executed to reveal categories to which each article was suitably assigned for ease of future assessment (refer to **Table 1**); “*New Development (N)*,” “*Psychometric Analysis (P)*,” and “*Translation (T)/Adaptation (A)*,” or a combination thereof. Each paper was assigned to the appropriate criteria; “*Development”* if the report’s main purpose was to produce an unprecedented tool, “*Psychometric Analysis”* if the explicit objective was to assess the reliability and validity of said tool, and “*Translation and/or Adaptation”* for all studies that in any way translated or altered a tool to suit a specific population, culture, and/or nation. Overall (**Table 2**), 36.8% of the studies aimed to merely psychometrically evaluate a pediatric sleep tool, while 9% additionally translated it. 24.3% of the studies aimed to independently translate while 4.2% additionally adapted their tool. As for lone adaptations, there were 4.2% of studies that performed this, while 18.8% created an entirely new tool. 1.4% of the studies conducted both a new tool development and translation and alike, 0.7% of studies adapted their new tool to particular population, culture, or other.

**Table 2:** Overview of psychometric analyses performed.

Tool acronym	NPTA	in Spruyt et al	Sleep categories	Factor analysis	Reliability analyses	Validity analyses	Confirmatory analysis	ROC	Normative values or cutoffs	Clinical classification	Specific population
**AIS** (5)	P		quality	structure	test-retest; internal	convergent/discriminant		yes; a total score ≥7		original AIS developed per ICD-10	DSM-IV-TR diagnosis of insomnia by interview
**ASHS** (6)	P	yes	regularity, hygiene, ecology,	structure	internal	convergent/discriminant	confirmatory				
**ASHS** (7)	P	yes	regularity, hygiene, ecology,		test-retest, internal	construct; convergent/discriminant					insomnia per DSM-IV-TR
**ASHS** (8)	PT (Farsi)	yes	regularity, hygiene, ecology	structure	test-retest, internal	convergent/discriminant	confirmatory				
**ASHS** (9)	PT (Persian)	yes	regularity, hygiene, ecology	structure	test-retest, internal	content; construct	confirmatory				
**ASQ** (10)	N		quality, sleepiness			face				ICSD	
**ASWS** (11)	P	yes	quantity, hygiene	structure	internal	content; construct	confirmatory				
**ASWS** (12)	P	yes	quantity, hygiene	structure	internal	construct					
**BEARS** (13)	PT (Spanish)	yes	quantity, quality, sleepiness			criterion					ICD-10 diagnoses assigned to these children, prior to the commencement of the parent group intervention were: F90, F98.2, F93.3, F80.1, F93.0, Z62
**BEDS** (14)	A	yes	quantity, quality, hygiene, ecology		test-retest; internal	construct; convergent/discriminant					Down syndrome
**BISQ** (15)	T (Spanish)	yes	quantity, hygiene		test-retest; interrater/observer	content; construct					
**BRIAN-K** (16)	N		regularity, hygiene,	structure	internal	content; construct					
**CAS-15** (17)	P		quality	structure	test-retest; internal; interrater/observer	construct; criterion; convergent/discriminant		yes; a score ≥32			
**CBCL** (18)	P	yes	quantity, quality,sleepiness		test-retest	convergent/discriminant					patients were diagnosed with sleep disorders according to ICSD-2
**CCTQ** (19)	T (Turkish)		quantity, regularity		internal	content					
**CCTQ** (20)	P		quantity, regularity		test-retest; internal	criterion					
**CCTQ** (21)	PT (Chinese)		quantity, regularity		test-retest. internal	content; construct					
**CRSP** (22)	P		quantity, quality, sleepiness, hygiene	structure		content; construct	confirmatory				
**CRSP** (23)	N		quantity, quality, sleepiness, hygiene		internal	construct; criterion; convergent/discriminant					
**CRSP** (24)	P		quantity, quality, sleepiness, hygiene	structure	test-retest; internal	construct; criterion; convergent/discriminant	confirmatory				
**CRSP** (25)	PT		quantity, quality, sleepiness, hygiene	structure	internal	convergent/discriminant	confirmatory		mean (SD)/n(%)		
**CRSP-S** (26)	P		sleepiness	structure	test-retest; internal	construct; convergent/discriminant	confirmatory				
**CSAQ** (27)	N		quantity, quality, sleepiness	structure	test-retest; internal; interrater/observer	content; construct; convergent/discriminant					
**CSHQ** (28)	P		quantity, quality, regularity, sleepiness, hygiene, ecology		test-retest	construct; criterion				original was designed to identify sleep problems based on ICSD-1	
**CSHQ** (29)	AT (Portuguese)		quantity, quality, regularity, sleepiness, hygiene, ecology	structure	test-retest; internal	convergent/discriminant				original was designed to identify sleep problems based on ICSD-1	
**CSHQ** (30)	P		quantity, quality, regularity, sleepiness, hygiene, ecology	structure						original was designed to identify sleep problems based on ICSD-1	
**CSHQ** (31)	P		quantity, quality, regularity, sleepiness, hygiene, ecology	structure	test-retest; internal	content; construct	confirmatory			original was designed to identify sleep problems based on ICSD-1	
**CSHQ** (32)	P		quantity, quality, regularity, sleepiness, hygiene, ecology	structure	internal	content; construct	confirmatory			original was designed to identify sleep problems based on ICSD-1	
**CSHQ** (33)	T (Dutch)		quantity, quality, regularity, sleepiness, hygiene, ecology	structure	test-retest; internal; interrater/observer		confirmatory			original was designed to identify sleep problems based on ICSD-1	
**CSHQ** (34)	T (Dutch)		quantity, quality, regularity, sleepiness, hygiene, ecology	structure	internal		confirmatory		a mean total CSHQ score of 41.9±5.6	original was designed to identify sleep problems based on ICSD-1	
**CSHQ** (35)	A		quantity, quality, regularity, sleepiness, hygiene, ecology		internal	convergent/discriminant				original was designed to identify sleep problems based on ICSD-1	allergic rhinitis
**CSHQ** (36)	A		quantity, quality, regularity, sleepiness, hygiene, ecology	structure	internal					original was designed to identify sleep problems based on ICSD-1	autism spectrum disorder
**CSHQ** (37)	P		quantity, quality, regularity, sleepiness, hygiene, ecology	structure	internal	criterion				original was designed to identify sleep problems based on ICSD-1	
**CSHQ** (short) (38)	A		quantity, quality, regularity, sleepiness, hygiene, ecology		internal	convergent/discriminant	confirmatory	yes; a total CSHQ score of ≥ 24		original was designed to identify sleep problems based on ICSD-1	clinical samples diagnoses based on the DSM-IV: pervasive developmental disorders, attention-deficit and disruptive behavior disorders, anxiety disorders; depressive disorders, and others and also without psychiatric disorder
**CSHQ** (39)	PT (German)		quantity, quality, regularity, sleepiness, hygiene, ecology	structure	test-retest; internal	content		yes; per subscale provided		original was designed to identify sleep problems based on ICSD-1	sleep disorders per ICSD II
**CSHQ** (40)	T (Portuguese)		quantity, quality, regularity, sleepiness, hygiene, ecology	structure	test-retest; internal	face				original was designed to identify sleep problems based on ICSD-1	
**CSHQ** (41)	PT (Spanish)		quantity, quality, regularity, sleepiness, hygiene, ecology	structure	test-retest; internal	face; content; construct				original was designed to identify sleep problems based on ICSD-1	
**CSHQ** (42)	T (Persian)		quantity, quality, regularity, sleepiness, hygiene, ecology	structure	test-retest; internal	face; content; construct; convergent/discriminant				original was designed to identify sleep problems based on ICSD-1	
**CSHQ** (43)	T (Portuguese)		quantity, quality, regularity, sleepiness, hygiene, ecology		test-retest; internal	content		yes; a cutoff total score of 44		original was designed to identify sleep problems based on ICSD-1	ICSD II for Sleep Related Breathing Disorder, Parasomnia, Behavioral Sleep Disorder
**CSHQ** (short) (44)	A		quantity, quality, regularity, sleepiness, hygiene, ecology			convergent/discriminant		yes; a cutoff total score of 30		original was designed to identify sleep problems based on ICSD-1	
**CSHQ** (14)	P		quantity, quality, regularity, sleepiness, hygiene, ecology		internal	construct; convergent/discriminant				original was designed to identify sleep problems based on ICSD-1	Down syndrome
**CSM** (45)	T (Polish)		regularity, sleepiness		internal	content; construct		accumulated percentile distribution			
**CSRQ** (46)	T (English)	yes	quantity, regularity, sleepiness	structure	internal		confirmatory				
**CSRQ** (47)	P		quantity, regularity, sleepiness			criterion		yes; ≥35; optimal sensitivity : 27.5; optimal specificity: 50.5			
**CSWS** (48)	P	yes	quantity, regularity	structure	test-retest; internal	content; construct	confirmatory				children with Sleep-Onset Association Problems per ICSD
**DBAS** (49)	T (German)		quantity, quality, regularity	structure	internal	content	confirmatory				
**DBAS** (50)	P		quantity, quality, regularity	structure	test-retest; internal	content					
**ESS** (51)	PT (Tamil)	yes	sleepiness	structure	internal	face; content; construct	confirmatory		>11 = excessive daytime sleepiness; 11-14 = moderate and >15 = high		
**ESS** (52)	P	yes	sleepiness		internal	convergent/discriminant		yes. cutoff score of 6			
**ESS-CHAD** (53)	P	yes	sleepiness	structure	test-retest; internal	construct; criterion					
**FoSI** (54)	PA		quality	structure	internal	convergent/discriminant	confirmatory				
**I SLEEPY** (55)	N		quality, sleepiness			criterion		yes; those endorsing three or more symptoms or complaints on the questionnaires			
**IF SLEEPY** (55)	N		quality, sleepiness			criterion		yes; those endorsing three or more symptoms or complaints on the questionnaires			
**I'M SLEEPY** (55)	N		quality, sleepiness			criterion		yes; those endorsing three or more symptoms or complaints on the questionnaires			
**ISI** (5)	P		quality	structure	test-retest; internal	criterion; convergent/discriminant		yes; a total score ≥9		partially diagnostic criteria of insomnia in DSM-IV	DSM-IV-TR diagnosis of insomnia by interview
**ISI** (56)	T (Swedish)		quality		internal	criterion				partially diagnostic criteria of insomnia in DSM-IV	chronic pain
**ISI** (57)	T (German)		quality	structure	internal	convergent/discriminant	confirmatory			partially diagnostic criteria of insomnia in DSM-IV	
**JSQ** (58)	P		quantity, quality, regularity, sleepiness, hygiene	structure	internal	content	confirmatory	yes; 80 for total score	standardized T scores by age and gender; 50.00 ± 10.00		
**JSQ** (preschool) (59)	P		quantity, quality, regularity, sleepiness, hygiene	structure	internal	face; criterion		yes; cutoff 84	standardized T scores by age and gender; 50.00 ± 10.00		
**LSTCHQ** (60)	N		quantity, regularity, sleepiness, hygiene, ecology		test-retest	face; content; construct					
**MCTQ** (61)	N	no, therefore added here	regularity								
**MEQ** (62)	T (Italian)		regularity, sleepiness	structure	internal	content					
**MEQ** (63)	P		regularity, sleepiness	structure	internal	convergent/discriminant					
**aMEQ** (64)	PT (European Portuguese)		regularity, sleepiness		internal	face; content			mean ± 1SD, percentiles 10 and 90, and the less restrictive percentiles 20/80; cut-points for the males and females		
**aMEQ-R** (65)	PA		regularity, sleepiness		internal	content; criterion; convergent/discriminant			aMEQ (≤45 and ≥60); aMEQ-R (≤23 and ≥33)		
**MESC** (66)	P	yes	regularity, sleepiness	structure	internal	convergent/discriminant	confirmatory				
**MESSi** (67)	PT (Turkish)		regularity, sleepiness	structure	internal	face; content; convergent/discriminant	confirmatory				
**MESSi** (68)	P		regularity, sleepiness		internal	convergent/discriminant	confirmatory				
**My Sleep and I** (69)	P		quantity, hygiene, ecology	structure	internal	convergent/discriminant	confirmatory				
**My children's sleep** (69)	P		quantity, hygiene, ecology	structure	internal	convergent/discriminant	confirmatory				
**NARQoL-21** (70)	NT (English)		quality, sleepiness	structure	test-retest; internal;	content; construct; convergent/discriminant	confirmatory	yes; a NARQoL-21 score below 42			diagnostic criteria for narcolepsy according to ICSD-3
**NSD** (71)	NA		quality								Asthma per Global Initiative for Asthma classification
**NSS** (72)	AT (Chinese)		sleepiness	structure	internal	face; content; convergent/discriminant					ICSD-3 criteria
**OSA Screening Questionnaire** (73)	N		quality			face; content					Down syndrome
**OSA-18 Questionnaire** (74)	T (Chinese)		quality	structure	test-retest; internal	construct; convergent/discriminant	confirmatory	yes; cutoff scores ranging from 55 to 66			OSA per ICSD 2
**OSA-18 Questionnaire** (75)	T (Chinese)		quality		test-retest; internal	construct; criterion					
**OSA-18 Questionnaire** (76)	T (Dutch)		quality		test-retest; internal	convergent/discriminant					craniosynostosis
**OSA-18 Questionnaire** (77)	T (Greek)		quality		test-retest; internal	criterion					
**OSA-18 Questionnaire** (78)	T (Portuguese)		quality		internal	convergent/discriminant					
**OSA-18 Questionnaire** (79)	T (Spanish)		quality	structure	test-retest; internal; interrater/observer	construct; convergent/discriminant					
**OSA-5 Questionnaire** (short) (80)	A		quality	structure	internal	content	confirmatory				
**OSD-6 QoL Questionnaire** (81)	T (Greek)	yes	quality		test-retest; internal	criterion					
**oSDB and AT** (82)	N		quality, treatment		internal	face; content; construct; criterion					
**OSPQ** (83)	N		quality, regularity, sleepiness	structure	test-retest; internal	face	confirmatory		the cutoffs for the 95th percentile (T-score of 70) by sex and age		
**PADSS** (84)	N		quality	structure	test-retest; internal	face; construct		yes; cutoff for the overall scale was located at 13/14			sleepwalking or sleep terror per ICSD
**PDSS** (85)	T (Brazilian Portuguese)		quantity, regularity, sleepiness		test-retest; internal	content					
**PDSS** (86)	T (Japanese)		quantity, regularity, sleepiness	structure	test-retest; internal	content					
**PDSS** (87)	T (Turkish)		quantity, regularity, sleepiness	structure	internal	content; construct	confirmatory				
**PDSS** (88)	P		quantity, regularity, sleepiness		internal	construct	confirmatory				
**PDSS** (89)	PAT (Russian)		quantity, regularity, sleepiness	structure	test-retest; internal	face; content	confirmatory				
**Pediatric Sleep CGIs** (90)	N		quantity, hygiene, ecology			convergent/discriminant				elements of insomnia as defined by the ICSD	Autism Spectrum Disorders
**PedsQL(fatigue scale)** (91)	AT (Arabic)		sleepiness		internal	content; construct; convergent/discriminant					cancer
**PedsQL (fatigue scale)** (92)	AT (Chinese)		sleepiness	structure	internal	content; construct; criterion	confirmatory				acute leukemia
**PedsQL(fatigue scale)** (93)	PT (Brazilian Portuguese)		sleepiness	structure	internal	construct; convergent/discriminant	confirmatory				cancer
**PISI** (94)	P		quality	structure	test-retest; internal	content; construct; convergent/discriminant	confirmatory			items per group consensus regarding the following ICSD-II general insomnia criteria	
**PNSSS** (95)	P		ecology		interrater					assess five of the AAP recommendations related to sleep practices	
**PosaST** (96)	T (Brazilian Portuguese)		quality		internal	criterion		yes; using the cumulative score ≥2.72 of the original scale			
**PPPS** (97)	P		quantity; regularity, sleepiness, hygiene		internal						
**P-RLS-SS** (98)	N		quality			face; content					including also ADHD subgroup per DSM-IV criteria
**PROMIS** (99)	P		quality, regularity, sleepiness		internal	face; content					
**PROMIS** (100)	P		quality, regularity, sleepiness	structure		content	confirmatory				
**PROMIS** (101)	P		quality, regularity, sleepiness	structure	internal	content; construct	confirmatory				
**PROMIS** (102)	PA		quality, regularity, sleepiness			content					
**PSIS** (103)	P		quality, regularity		internal	content; construct					child psychopathology and functioning per DSM-IV-TR
**PSQ** (104)	P		quality		internal						obese adolescents undergoing bariatric surgery
**PSQ** (105)	T (Turkish)		quality		internal	content; construct				items similar DSM-IV	
**PSQ** (106)	T (Spanish)		quality	structure				yes; cutoff score >0.33			
**PSQ** (107)	T (Malay)		quality		test-retest; internal	face; content					
**PSQ** (108)	P		quality			criterion		yes; original 0.33 and AHI>1.5			
**PSQ** (109)	P		quality			face; content		yes; cutoff of 0.72–0.76.			asthma per ICD 9
**PSQ** (110)	PT (Chinese)		quality	structure	test-retest	content; construct					
**PSQ** (111)	T (Thai)		quality		test-retest; internal	face; content		yes; a cutoff of >0.33			
**PSQ** (112)	P		quality					yes; a cutoff value of seven points			
**PSQ** (113)	PT (Portuguese)	yes	quality		test-retest; internal	face; content					
**PSQ** (114)	PT	yes	quantity, quality, regularity	structure	test-retest; internal	face; construct	confirmatory				
**PSQI** (115)	T (Brazilian Portuguese)	yes	quantity, quality, regularity	structure	test-retest; internal	content	confirmatory				
**PSQI** (116)	P	yes	quantity, quality, regularity	structure	internal	content; convergent/discriminant	confirmatory				
**RLS** (117)	NP		quality		test-retest; internal	face; content			calculated RLS index (difference in score between 14 day time points); one control subject had a higher index value (14) than two RLS-diagnosed (10 and 13)	criteria for children established by the International Restless Legs Syndrome study group	
**SDIS** (118)	P	yes	quantity, quality, sleepiness			convergent/discriminant					insomnia per ICSD-2 or ICSD-3
**SDPC** (119)	P		quantity, quality, sleepiness			content					cancer
**SDSC** (120)	T (Chinese)	yes	quantity, quality, sleepiness	structure	internal	construct	confirmatory			original SDSC fits ASDC	
**SDSC** (121)	T (French)	yes	quantity, quality, sleepiness	structure	test-retest; internal; interrater/observer	construct; convergent/discriminant	confirmatory		T-score >70	original SDSC fits ASDC	
**SDSC** (122)	T (Persian)	yes	quantity, quality, sleepiness		internal	construct; convergent/discriminant				original SDSC fits ASDC	
**SDSC** (14)	P	yes	quantity, quality, sleepiness		internal	construct; convergent/discriminant				original SDSC fits ASDC	Down syndrome
**SDSC** (123)	P	yes	quantity, quality, sleepiness		internal	construct; convergent/discriminant				original SDSC fits ASDC	neurocritical care acquired brain injury
**SDSC** (124)	P	yes	quantity, quality, sleepiness				confirmatory				ADHD
SDSC* (125)	N		quantity, quality, regularity, sleepiness	structure	internal	content				ICSD 2 as reference	
**SHI** (126)	T (Turkish)		quantity, quality, sleepiness	structure	test-retest; internal	construct	confirmatory				major depressive disorder per DSM-IV criteria
**SHIP** (127)	N		quantity, regularity, sleepiness		internal	content; construct; criterion; convergent/discriminant					chronic headache per International Headache Classification
**Sleep Bruxism** (128)	N		quality								
**SNAKE** (129)	N		quantity, quality, regularity, sleepiness, hygiene, ecology	structure	test-retest; internal	construct; convergent/discriminant	confirmatory		T-score and percentage rank for raw score per factor	per ICSD-2	severe psychomotor impairment
**SQI** (5)	P		quality	structure	internal	convergent/discriminant		yes; total score ≥5			DSM-IV-TR diagnosis of insomnia by interview
**SQ–SP** (130)	P	yes	quantity, quality, sleepiness,	structure	test-retest; internal	construct; convergent/discriminant	confirmatory				individuals with intellectual disability
**SQS-SVQ** (131)	AT (Turkish)		quantity, regularity, ecology	structure	test-retest; internal	criterion	confirmatory			sleep quality items comparable to DSM IV insomnia criteria	
**SRSQ** (132)	N		quantity, quality, regularity, sleepiness		test-retest; internal	content		yes; a cutoff of 17.3			
**SSR** (133)	T (Spanish)		quality, regularity, sleepiness	structure	internal	construct; convergent/discriminant	confirmatory			original items per ICSD	
**SSR** (43)	T (Portuguese)		quality, regularity, sleepiness		internal	content				original items per ICSD	
**SSSQ** (134)	N		quantity, regularity		test-retest	criterion					
**STBUR** (135)	N		quality	structure				yes; 10.40 (1.37–218.3) for 5 items			
**STQ** (136)	P		quantity, regularity			convergent/discriminant					
**The Children's Sleep Comic** (137)	N		quantity, quality, regularity, sleepiness, hygiene			content; construct				ICSD-2	
**The Children's Sleep Comic** (138)	P		quantity, quality, regularity, sleepiness, hygiene		internal	content; convergent/discriminant		yes; a total intensity of sleep problem score of 9	stanine value (5±2), percentile rank and relative frequency for the raw intensity of sleep problem score	ICSD-2	
**TuCASA** (139)	AT (Portuguese)	yes	quality		internal	content; convergent/discriminant					
**YSIS** (140)	NT (English)		quality	structure	test-retest; internal	face; content; construct; convergent/discriminant	confirmatory	yes: Normal ∶< 22 (< 70th percentile); Mild insomnia ∶ 22 (70th percentile)−25; Moderate insomnia/clinical insomnia ∶ 26 (85th percentile)−29; Severe insomnia/clinical insomnia ∶≥ 30 (95th percentile		based on ICSD-3 [12] and DSM-V [13] diagnostic criteria	

### Study Characteristics

The structural organization and publication features of each study are detailed in **Table 1**. In the **Appendix** are the acronyms for each tool reviewed. Since the 2011 Spruyt review on pediatric diagnostic and epidemiological tools, approximately 144 “tool”-studies have been published. The focus into pediatric tool evaluation peaked in 2014 where 16.7% of all studies were conducted, closely followed by 2017 (13.9%), and 2016 and 2019, each at 13.2% as well as 2015 at 12.5%. As for the remaining years of this decade, between 2010 and 2014, 2018 , the percentage of total studies published ranged from 0.7%–9.7% (n=1–10) per year. Over a third of the total studies were published in Europe (38.9%), followed by North America (25%), Asia (18.1%), Middle East (2.8%), South America (7.6%), Australia and Oceania (6.3%), and the United Kingdom (1.4%).

Across all 144 studies evaluated, it was evident that sleep tools were predominantly developed and evaluated for a combination of children and adolescents between the ages of 6–18 years (27.1%), followed closely by tools for adolescents 13–18 years at 22.2% and children 6–12 years alone at 16.7%. Only 10 studies covered the 0–18 years age range, and one did not define its range (82). Meanwhile, only 5.6% of all the studies assessed tools for preschool-aged children (2–5 years) alone and 1.4% for infants (0–23 months) alone. As for the studies remaining, a combination of age ranges was investigated with the most predominant combination being both preschool children and children (ages of 2–12 years) at 8.3% of the total studies. The lesser frequent combinations of age ranges for which tools were assessed in these studies, ranged from 0.7–7.6% per combination.

As for the sample size, this ranged between 20 and 11,626 children inclusive of adult (6–13) participants across all publications, where 15.6% of all studies used a sample size >1,000 participants large (**Table 2**). Of these study samples, approximately 46.5% of respondents were parents, 41% were self-report, and 11.1% either a combination of experts, children, mothers, and parents. For two, the respondent is primarily a professional (17, 95).

#### Sleep Categories

As exemplified in **Table 2**, the overall focus of these studies was overwhelmingly directed at tools measuring the quality of sleep or identification of sleep pathologies in all pediatric age classifications (68.1%), followed by the levels of sleepiness (55.6%) and duration of sleep (48.6%). Various secondary coobjectives of these studies were to investigate tools measuring the sleep regularity (46.5%) and sleep hygiene practices (29.2%). Rarely but in existence, was the singular assessment of sleep ecology and treatment around sleep pathologies at a frequency of 21.5% and 0.7%, respectively. About 19 studies (13.2%) queried simultaneously nearly all categories (except treatment).

#### The 11 Steps

Regarding the psychometric evaluation step-by-step guide proposed by Spruyt (2, 3), less than half the required 11 steps (chiefly 1, 2, 6, 8, and 9 were done) were fulfilled across all studies. Steps 3 and 10 were often not reported (i.e., 84.7% and 63.2%, respectively). Three studies reported all steps (2.1%), three only lack step 11 (2.1%), and four (2.8%) only lack steps 10 and 11. The most common combination of steps (7.7%) reported are 1, 2, and 4 joined with 5, 6, 7, 8, 9 or 5, 6, 8, 9 or 6, 7, 8, 9, 10. After a decade, only 18 papers (12.5%) reported some form of norms. An in-depth description of the steps fulfilled is described in the categorically-divided (per purpose, see Methods) results below.

### Tools Newly Developed

According to our search criteria, a total of 27 novel pediatric sleep tools were developed between 2010 and 2020 (refer to **Table 2** and shaded). Of these, approximately eight were published in Europe (29.6%), eight in North America (29.6%), four in Asia (14.8%), three in South America (11.1%), two in Australia and Oceania (7.4%), and two in the United Kingdom (7.4%). The majority were developed for child-adolescent age ranges (66.7%), while one for preschool children (2–5 years) and one for all three aforementioned ages (2–18 years). All newly developed tools possessed a multipurpose objective, most of which assessed sleep quality (77.8%), followed by the assessment of sleepiness (51.9%) and sleep regularity (41.7%) and sleep quantity (41.7%), while more rarely assessing hygiene (25%), ecology (12.5%), and treatment (4.2%).

In addition, three tools being newly created are an English translation of the NARQoL-21 (70) and YSIS (140), and also an adaptation, the nighttime sleep diary (NSD) (71). The latter being a diary adapted to monitor nighttime fluctuations in young children with asthma.

Only two tools were developed according to the 11 aforementioned steps required for psychometric validation of a tool; the NARQoL-21 (70) and SNAKE (129) (refer to **Table 2**). One other tool, OSPQ (83) also developed normative scores for widespread usage while fulfilling most steps but steps 3 and 9. Whereas the CSAQ (27) fulfilled all steps except step 11, and the BRIAN-K (16), PADSS (84), and SDSC* (125) except steps 10 and 11. The outstanding tools were mostly absent of steps 5, 7, 8, 9, and 10. For the newly developed diary, NSD (71) steps 1–6 were fulfilled.

Almost half of the tools queried general sleep problems (41.7%). Twenty-five percent aimed at surveying sleep disordered breathing. While others such as sleep bruxism (128), PADSS (84), P-RLS-SS (98), RLS (117), NARQoL-21(70), YSIS (140), and NSD (71) focused on a specific sleep problem (16.7%). Tools aimed at investigating sleep complaints in children with (developmental) disabilities are besides NSD (71), the OSA Screening Questionnaire (73), Pediatric Sleep CGIs (90), SHIP (127), and SNAKE (129).

### Tools Translated

In total, 35 out of the total 144 studies primarily aimed to translate an existing tool alone (refer to **Table 2**). Namely, 17 tools have been translated: BISQ (15), CCTQ (19), CSHQ (29, 33, 34, 40–43), CSM (45), CSRQ (46), DBAS (49), ISI (56, 57), MEQ (62), OSA-18 (74–79), OSD-6 (81), PDSS (85–87), PosaST (96), PSQ (105–107, 110, 111, 113), PSQI (115), SDSC (120–122), SHI (126), and SSR (43, 133). The most frequently translated tools were: OSA-18 (17.1%), CSHQ (14.3%), and PSQ (11.4%). The most common translation was to Portuguese (n=4), Spanish (n=4), and Turkish (n=4), followed by Brazilian Portuguese (n=3), Chinese (n=3), and Dutch (n=3). Less often, tools were translated to German, Persian, and Greek as well as English, Italian, Polish, Swedish, Japanese, French, Malay, and Thai. Again, primarily tools for child/adolescent age ranges as parental reports have been translated. Of these, the main categorical foci, and often overlapping, were sleep quality (77.1%), quantity (48.6%), and sleepiness (48.6%).

When ranked from most to least prevalent step, apart from steps 1 and 2, we found: step 8 (97.1%), step 4 (91.4%), step 9 (88.6%), step 6 (85.7%), step 5 (57.1%), step 7 (51.4%), and step 10 (34.3%) being performed across the studies. The CSHQ (34) and SDSC (120, 121) included norm development (step 11). Step 3 is missing in all translations. Only the translation of the SDSC fulfilled nearly all steps with (121) missing step 3 and (120) missing steps 3 and 9. Receiver Operator Curve (ROC) analyses were performed in five : OSA-15 (74), PosaST (96), PSQ (106, 111), and CSHQ (43).

### Tools Adapted

Moreover, six studies (see **Table 2**) specifically aimed to adapt a tool from a preexisting one, most notably the Children’s Sleep Habits Questionnaire (CSHQ) (66.7%), among these a shortened version and infant adaptation, along with the BEDS (14) (16.7%) adapted toward children with Down syndrome, and the OSA-18 Questionnaire (16.7%), which was also shortened [toward OSA-5 (80)] to suit the sample of interest. Although the number of items may have changed, no substantial changes to the answer categories could be noted. Only 33.3% reported steps 3, 4, 5, 7, 10 yet steps 6, 8, 9 were analyzed in 83.3%. None developed norms. In two studies (38, 44) ROC analyses were pursued for the CSHQ.

#### Tools Adapted and Translated

Six studies adapted and also translated existing tools (see **Table 2**): CSHQ (29), PedsQL (91, 92), SQS-SVQ (131), TuCASA (139), and NSS (72). The CSQH and TuCASA were adapted and translated to Portuguese, the PedsQL to Arabic and Chinese, while SQS-SVQ to Turkish and NSS to Chinese. The adaptations involved an infant version of CSHQ and child-sample for NSS, the PedsQL to children with cancer and acute leukemia, and the TuCasa was adapted toward children of low socioeconomic status. Regarding the SQS-SVQ it was modified based on personal communication with the authors of the original version. That is, four items were added.

For these tools Steps 3 and 11 were not performed, while Steps 8 and 9 were performed in all. About half (50%) did steps 5, 6, and more than half step 7 (66.7%) and less than half did step 10. Some aspects of step 4 were inconsistently applied across 83.3% of the studies (e.g., expert perspective).

### Tools Psychometrically Evaluated

Approximately 53 studies were published that focused solely on psychometric evaluation of questionnaires between 2010 and 2020 (refer to **Table 2**). Of these, commonly investigated were CSHQ (11.3%), CRSP, and PSQ (each 7.5%), followed by SDSC and PROMIS (each 5.7%). The greatest number were printed in 2014 (15.1%), as well as 2018 and 2019 (each 13.2%) and 2015, 2016, 2017 (each 11.3%), and a lesser number of instruments were evaluated in the other years. In terms of location, the majority were published in North America (43.4%) followed by Europe (22.6%) and Asia (18.9%), Australia and Oceania (11.3%), and the South America (3.8%). Especially tools for adolescent age ranges (34%) were psychometrically evaluated, followed by child-adolescent age range (22.6%). 9.4% involved tools for preschoolers (2–5 years) and 15.1% are for child (6–12 years) alone. The remainder are combinations: preschooler child (3.8%), preschool to adolescent (9.4%), and all (0–18 years; 3.8%).

Ranked on sleep category, the tools examined: 64.2% sleep quality; 58.5% sleep quantity; 47.2% sleep regularity; 58.5% sleepiness; 35.8% sleep hygiene, 20.8% sleep ecology but none for treatment. Among all 53-instrument validations, none adhered to all eleven recommended steps of tool evaluation. Besides steps 1 and 2, especially steps 9 (90.6%) and 8 (75.5%), 6 (64.2%) have been reported upon psychometrically evaluating tools, and less common have been steps 7 (54.7%), 10 (41.5%), and 4 (34%). Least common in psychometric screening were steps 5 (13.2%), 3 (13.2%), and again 11 (15.1%). ROC analyses were performed in 11 studies (20.8%): ESS (52), AIS and SQI (5), JSQ (58, 59), PSQ (108, 109, 112), CAS-15 (17), CSRQ (47), and Comics (138). Almost fulfilling all steps were: CAS-15 (Goldstein et al., 2012) and Comics (137, 138).

#### Tools Psychometrically Evaluated and Adaptations

Three tools underwent evaluation but were simultaneously modified: FoSI was adapted for adolescents (54), and a reduced itemset was suggested for aMEQ-R (65) and PROMIS (102).

#### Tools Psychometrically Evaluated and Translated

In addition to the 53 instruments validated, there were 13 studies flagged that additionally translated their respective tools (refer to **Table 2**); the ASHS to Persian, the BEARS to Spanish, CCTQ to Chinese, the CSHQ to German and Spanish, the ESS to Tamil, the MEQ to European Portuguese, the MESSi to Turkish, the PSQ to Chinese, Portuguese and French, and the PedsQL to Brazilian Portuguese. Step 9 was performed in all studies, closely followed by steps 4, 6, and 8 (93.3% each). Step 7 (69.2%) and 5 (53.8%) and 10 (46.2% each) were not as frequently pursued. Again, steps 3 and 11 (15.4%) were nearly absent in the psychometric evaluation. Of these, the ESS (51) underwent all steps.

#### Tools Psychometrically Evaluated, Translated With Adaptations

The Russian version of the PDSS (89) did not report step 3, but executed to a certain extent all the steps to psychometrically evaluate a translated tool to its population. Based on the advice of the area specialist and the focus group of children questions #3 (Trouble getting out of bed in the morning), 4 (Fall asleep/drowsy during class), 7 (Fall back to sleep after being awakened), and 8 (Usually alert during the day (reverse coded)) were modified for better understanding.

### Some Extra Remarks

#### Translations of Tools

Although the studies reported here are English papers, popular translations are Chinese, Portuguese, Spanish, and Turkish. The CSHQ, PSQ, and OSA-18 were the most frequently translated tools.

#### Tools With Norm Scores

Psychometric studies of particular interest are those that developed normative values or clinical/community cutoff scores for widespread usage, of which there were overall 18. Norms have been developed for CAS-15 (17), ESS (51, 52), JSQ (58, 59), SDSC (120, 121), CSHQ and CRSP (25, 34), CSRQ (47), MEQ (64, 65), NARQoL-21 (70), OSPQ (83), PSQ (108), SNAKE (129), Comic (138), and YSIS (140) (refer to **Table 2**).

The CAS-15, PSQ, CSRQ, and ESS studies provided “normative” ROC cutoff scores, with the Krishnamoorthy et al. (51) providing cutoffs for moderate and high excessive sleepiness.

Population-based norms were developed for preschoolers and school-aged children of JSQ. Average T-scores for all as well as for boys/girls in age bands of 2–3, 4, 5–6 years separately are available for each subscale: restless legs syndrome, sensory; obstructive sleep apnea syndrome; morning symptoms; parasomnias; insomnia or circadian rhythm disorders; daytime excessive sleepiness; daytime behaviors; sleep habit; insufficient sleep; and restless legs syndrome, motor. For school-aged median T-scores are available for 1^st^–2^nd^, 3^rd^–4^th^,5^th^–6^th^ grade per the following subscales: restless legs syndrome, sleep disordered breathing, morning symptoms, nighttime awakenings, insomnia, excessive daytime sleepiness, daytime behavior, sleep habit, and irregular/delayed sleep phase.

Regarding the SDSC, French (France and French speaking Switzerland) as well as Chinese T-scores are available. The Chinese study reports average T-scores per the subscales sleep–wake transition disorders; disorders of initiating and maintaining sleep; disorders of excessive somnolence; disorders of arousal; sleep hyperhidrosis; and sleep breathing disorders. Whereas the French study copied the approach of the original report, i.e., tabulated the full T-score range from 31 to 100 including marks for clinical ranges.

The CSHQ study aimed to validate the Dutch version of the tool for toddlers while developing norms due to the current inaccessibility of the CSHQ in this age group. Norm values were decidedly the mean total score in the sample population and while the factor-structure was unsupported, the normative score developed was still representative of the presence and severity of sleep problems in 25% of toddlers. Authors report the mean total score for lower/higher socioeconomic status, 2 and 3 year olds, girls and boys, yes/no problem sleepers. The authors similarly provided means and standard deviations for the 23 items of the CRSP.

The MEQ studies are comparable providing means and standard deviations as well as percentiles. Also percentiles are reported in the YSIS study.

For the NARQoL-21 a comparison was made with a validated health-related quality of life tool, and a cutoff of <42 was deemed as sensitive and specific, supplementary available are cutoff scores for differentiating between optimal and suboptimal quality of life.

T-scores for subscales by gender and age (5–7 and 8–10 years old) are provided for OSPQ: sleep routine, bedtime anxiety, morning tiredness, night arousals, sleep disordered breathing and restless sleep.

For SNAKE a t-distribution was generated for Disturbances going to sleep, Disturbances remaining asleep, Arousal disorders, Daytime sleepiness, and Conduct disorders for children in ages between 1 and 25 years old. For the Children’s Sleep Comic (ages 5 to 11) stanines were generated for the raw intensity of sleep problem score.

#### Tools With ROC Analyses

Twenty-eight (19.4%) studies reported ROC findings. This was primarily done for (refer to **Table 2**) CSHQ (n=4) and PSQ (n=5). That is, in 20% the ROC was calculated given clinical versus control/community samples, while in 48% of the papers a PSG parameter was used (e.g., apnea-hypopnea index, obstructive index). Another criterion was used in 32% of the cases (e.g., validated questionnaire, parental report, or optimal cutoff from original paper).

#### Papers With Questionnaires Available

In **Table 1**, the studies (32.6%) that printed or made available their questionnaire in supplementary files or appendix are shown.

#### Use of Classification Systems

Primarily the ICSD classification system was used to generate/mimic items for the following new tools: the Pediatric Sleep CGIs (90), RLS (117), SDSC* (125), SNAKE (129), the Children's Sleep Comic (137), and YSIS (140). When tools were psychometrically evaluated and/or translated/modified such as the CSHQ or the SDSC the classification system upon which their original items were generated remains.

#### Tools Used in Specific Populations

The SNAKE has been specifically developed for children with psychomotor disabilities, and hence serves as a good example of tool development. Whereas the vast majority of studies involved tools that are modifications or compilations, as well as a psychometric evaluation of the tool utility in an “atypical” population.

## Discussion

Since the 2011 Spruyt (2, 3) review, it has been encouraged that further psychometric validation is pursued for all questionnaires to develop a broader and more reliable range of tools. While “*tools do not need to be perfect or even psychometrically exceptional, they need to counterpart clinical decision-making and reduce errors of judgment when screening for poor sleep,*” suggested Spruyt (personal communication). This is done through the descriptive, iterative process of a tool protocol and often requires all steps of psychometric evaluation. Without this we have observed that tools rely on minor aspects of their psychometric validity for (clinical) application when this is often fallacious and nonspecific to the study population. Following the systematic review however, a dramatic increase in tool translations and adaptations has been observed which is to be irrefutably applauded. Nonetheless, it is important to develop standardized tests that are culture-free and fair in order to identify sleep issues across the board based on an unbiased testing process.

Twenty-seven new tools have been developed, while most of the papers published reported translations/adaptations or a psychometric evaluation of an existing tool. More than half of the tools queried general sleep problems. Irrespective of the infrequency of tools developed in categories like sleep ecology and treatment, there is an emerging need for further research into these areas given the environmental impact of technology on pediatric sleep in the 21^st^ century (141, 142).

The two new tools that underwent all 11 steps aimed at investigating sleep problems either in terms of a quality of life tool for narcoleptics (NARQoL-21) (70) or as a sleep disorder tool for children with severe psychomotor impairment (SNAKE) (129). Several other tools accomplished nearly all steps (see Tables: OSPQ, CSAQ, BRIAN-K, PADSS, SDSC*, NSD, and YSIS).

Since the 2011 review, tools for specific populations (e.g., in terms of ages, developmental disabilities, sleep pathologies) are still needed. Epidemiological tools assessing sleep in adolescents specifically have received some focus, where they were second in publication frequency. This dramatic influx of relevant research can be a result of the rising sleep-reduction epidemic in teenage populations influenced by biological, psychological and sociocultural factors. In addition, the investigation into the effects of sleep hygiene and ecology (143), which are heavily influenced by sociocultural phenomena, have slowly presented themselves across children and adolescents (6–18 years). With the introduction of technology at the forefront of childhood influence (144, 145), pediatric sleep habits and consequently quality is slowly gaining traction where studies flagged here are acknowledging the underlying weight of sleep hygiene on sleep quality and sleep quantity. Although at present, these tools are still demanding attention for further psychometric validation. An urgent call for tools with adequate psychometric properties is concluded in several recent reviews (146–148).

Especially assessing the factor structure of tools toward construct validation has been pursued, while other steps continue to be overlooked. Similarly, general tools to screen for sleep pathologies remain preponderant since the 2011 review. Alternatively, a file-drawer problem can be expected. Combined with the difficulty of finding a suitable journal to publish a tool validation study, this may lead to a skewed scientific literature toward commonly published and used tools. This is potentially echoed in atypical populations as seen by the influx of psychometric evaluations of existing tools. Undoubtedly, more studies are needed in an era where sleep is rapidly gaining public interest, and the need for a scientifically sound answer on the consequences of a “poor sleep” endemic is pressing.

Several tools pop out for diverse reasons. The first tool of note is the JSQ (58, 59) validated for Japanese children investigating sleep in a large population-based sample flagged by our search and developing normative values for this tool at a 99% confidence interval. This tool is notable in that given its statistical validity and reliability in a large population sample, the plausibility of this being mirrored in other cultures is possible. Important to note however, is that sleeping habits in Japanese children may vary greatly to those in western countries. Therefore, the changes in sociocultural sleep habits when adapting for other populations should be considered. Secondly, SNAKE the sleep questionnaire for children with severe psychomotor impairment underwent all 11 steps and was uniquely developed (hence not modified) for a specific population. More alike are needed (149). Thirdly, PADSS, and BRIAN-K both newly developed tools drew our attention because they examine arousal level and biological rhythm. Although the PADSS may need some further validation studies toward diagnosing, monitoring, and assessing the effects of treatment in arousal disorders in childhood particularly, it addresses the need for more specialized tools. Whereas the BRAIN-K being a modification of an adult version may benefit from additional psychometric evaluations beyond the current age range. Also, the FoSI, measuring fear, being based on the adult version assessing fear in a rural trauma-exposed sample (150) warrants further psychometric scrutiny. In contrast to others, the RLS (117) proposes a difference in scores between two time points 14 days apart to identify RLS-related symptoms. Lastly, addressing the need for tools allowing the child to express themselves regarding sleep is the Children's Sleep Comic, being an adapted version of the unpublished German questionnaire “Freiburger Kinderschlafcomic” and providing pictures for items and responses. Hence, pinpointing to the “un”published tools in the field and a welcomed child’s perspective regarding inquiring about sleep in an alternative way.

Adhering to the words of Spruyt, that instruments should be enhancing clinical decision-making and significantly reducing errors of judgment, the study by Soh et al. identified, developed, and abbreviated the OSA-5 questionnaire after recognising preexisting faults in the original 18-item version. It was identified that the OSA-18 was initially designed as a disease-specific quality of life tool that does not predict obstructive sleep apnea (OSA) symptoms consistent with the gold-standard PSG. Recently Patel et al. (151) scrutinized the accuracy of such clinical scoring tools. Additionally, the study by Soh et al. (80) acknowledged that there exists a lack of parental understanding of some items and their wording in the original instrument. As a result, the OSA-18 was abbreviated to 11-items and then to 5- so that ultimately it would “*perform better as a screening tool for use in triage and referral planning.”* Our review also revealed other tools addressing this sleep problem: I’m sleepy (55). While OSA is increasingly relevant in pediatric epidemiology due to the rise in obesity, parental knowledge of the condition and consequent treatment options is imperative. A recent 2017 study regarding the development of a questionnaire informing parents of this treatment was designed by Links et al. (82). The tool aims to alleviate parental conflict around the choice for or against this treatment in children and is a first in its approach as a questionnaire focusing on medical treatment decision making. Like the objectives of OSA-5, this tool is notable in that it aims to *“improve the quality and impact of patient and family decisions about OSA diagnosis and treatment”* (82). As part of the personalized/precision medicine era, the CAS-15 (17) and PROMIS-papers pop out. The CAS-15 is one of the few tools where the respondent is the professional. The PROMIS, although presented as a potential screening/diagnostic tool, recently underwent several psychometric evaluations. It involves an item bank of Patient Reported Outcomes Measurement, or better it is intended to measure the subject’s “view” of their health status (e.g. sleep). Although these patients reported outcome measures (PROM) adhere to the same psychometric characteristics as diagnostic/screening tools, the scope of a PROM is very different. Namely, PROMs allow the efficacy of a clinical “intervention” to be measured from the patients’ perspective. Unfortunately, these specific instruments have not undergone all steps, accordingly, they would benefit from further validation and possible cultural/linguistic adaptation to achieve a more widespread use in the future.

As for the majority of tools that lack the detailed mention above, there is need for comment on the gradually increasing recognition for disease-specific instruments or instruments for specific populations. Alternatively, measuring the severity of sleep conditions over the frequency is still much needed. It was observed by Spruyt that nearly all questionnaires up until the 2010 search, focused on the frequency of sleep problems, however since then, several tools have aimed to increase the specificity and sensitivity of sleep tools to the severity of common pediatric illnesses and specific age groups associated with them e.g. Down syndrome, Narcolepsy (148), infancy, etc. This specificity of condition severity and age may help to refine treatment measures and streamline clinical interventions.

Additionally, in contrast to our review in 2011, the studies reported here are English papers, although popular translations are Chinese, Portuguese, Spanish, and Turkish. That is, between 2010 and 2020 especially the CSHQ, PSQ, and OSA-18 were translated. This is likely an approximation due to the exclusion of non-English papers and of dissertations etc. In 2011, we observed that the development or modification of tools may not always evolve into a scientific paper.

Vis-à-vis fulfillment of psychometric criteria, preliminary and confirmative factor analysis methods have been included in the scope of, and completed in either partially or completely, most the studies which was lacking prior. Primarily construct and content validity *via* factor structure or item correlation, and Cronbach alpha statistics are noticed. Standardized scoring and item generation however, is still ill-managed as a requirement and is an important step in developing a diagnostic tool or adapting/translating an existing one. Nonetheless, generally, it can be said that much of the studies into tool-psychometrics deserve recognition for endeavoring to adhere to steps 1 through 11. But the overarching suggestion thus far, is to more thoroughly fulfill the facets of validation; i.e. content, convergence, discriminative, and criterion-related validity (steps 8 and 9), pilot questionnaires in the event of an adaptive change made (step 5), examine the underlying factors to ensure (uni)dimensional structure of a said tool (steps 7 and 10) and develop norms alongside cutoff scores (step 11). Furthermore, although several tools mimic classification systems a more thorough psychometric scrutiny thereof is still needed. As a consequence, to date, the vast majority of tools reflect an appraisal of the frequency of a sleep complaint.

Several limitations should be noted. We post hoc limited our flagged studies to only English language given that they reach the broader scientific community. Furthermore, several of the tools included are not 100% sleep tools (e.g. health related). In addition, our way of presenting being “New Development (N),” “Psychometric Analysis (P),” and “Translation (T)/Adaptation (A),” or a combination thereof, involved overlaps in descriptive analyses. Contrary to the original paper by Spruyt, this one did not apply searches in Dissertations and Theses, Google Scholar (Web crawling), ebooks and conference Sleep abstract books, and as a consequence might not be an exhaustive list of tools. Alternatively, studies involving app’s did “hit” our search terms yet were not retained during further screening toward our aims. Lastly, given that this is a systematic review we didn’t pursue a quality assessment of study designs investigating sleep tools. Nevertheless, in Spruyt et al. (2) each of the necessary steps are stipulated.

### Recommendations

It is recommended that future tools further the investigation into sleep hygiene, ecology [see (143)] and schedules of pediatric populations as this is becoming a highly relevant field of research upon the introduction of technology into sleeping habits and routines. The increasing prevalence of sleep deprivation in children (152–155) requires in depth discovery as to what damage or lack thereof is being done as a result of a 21^st^ century society.

In addition to this, it is suggested that pediatric tools should be further introduced and adapted or validated for reporting by children older than 8 years of age. Since there is evidence to suggest that children as young as eight years can report information critical to their own health, it is recommended that a large proportion of questionnaires be designed for children in this age category as well as parents (1). Conjunctional use of these however, is advised to develop any diagnosis.

Although several tools listed mimic classification systems, or were psychometrically evaluated in samples that underwent clinical diagnoses upon a classification system, there is still room for improvement. Combined with primarily convenience samples such as clinical referrals and lack of details on (at risk of being poor) sampling techniques, the internal and external validity of studies might be seriously jeopardized.

Sensitivity and specificity are key in differencing screening versus diagnostic tools. Yet also, the sample on which this difference is determined plays a key role, where the diagnostic tools chiefly aims at subjects believed to have the problem. Thus, screening tests are chosen toward high sensitivity while diagnostic tests are chosen toward high specificity (true negatives).

Lastly, caution is warranted upon a general positive score regarding reliability and validity assessment, and readers are advised to remain critical concerning the statistical techniques applied in the individual studies. Several recommendations for future tool development or evaluation have been listed in **Box 1**. Tool development and evaluation, as mentioned in the past is time and labor-intensive (2). In short, scientific copycats (i.e. replication studies) are needed!

Box 1 **Research agenda: a need for**

- Tools assessing sleep ecology, sleep routines/hygiene, regularity, treatment
- Psychometric evaluation of apps
- Tools for daytime sleep
- Tools per sleep pathology
- Tools for specific populations
- Tools sensitive and specific regards classification systems
- Tools adept to developmental changes
- Tools differentiating between school days and nonschool days
- Tools as a PROM, Patient-Reported Outcome Measures
- A venue to publish psychometric evaluations of tools
- Methodologic scrutiny regarding sampling (patient/population), statistical techniques, the aim(s), and type of study
- Availability of the tools published, especially translations
- Equal attention to all 11 steps; e.g. step 3 such as answer but also time format
- Replication studies
- Self-reporting tools for school-aged children
- Question and/or Response formats beyond frequency
- Sleep duration not being a categorical answer
- Caution regarding “child”-modifications of adult tools or applications beyond the intended age range
- Culture-free or fair tools
- Reviews and meta-analyses on criterion validity of subjective tools

## Author Contributions 

TS performed first search, extracted data, and wrote the first draft during her internship. Her work was updated, verified and finalized by KS.

## Conflict of Interest

The authors declare that the research was conducted in the absence of any commercial or financial relationships that could be construed as a potential conflict of interest.

## Abbreviations

AAP, American Academy of Pediatrics; ADHD, attention deficit hyperactivity disorder; ASDC, Association of Sleep Disorders Centers classification; DSM, Diagnostic and Statistical Manual of Mental Disorders; ICD, International Classification of Diseases; ICSD, International Classification of Sleep Disorders; PSG, polysomnography; RLS, Restless Legs Syndrome; ROC, Receiver Operating Characteristic curve.

## Appendix

### Appendix

**Table:**

**Tool acronym**	**Tool**
AIS	Athens Insomnia Scale
ASHS	Adolescent Sleep Hygiene Scale
ASQ	Auckland Sleep Questionnaire
ASWS	adolescent sleep wake scale
BEARS	Bedtime problems (B) Excessive daytime sleepiness (E), Awakenings During the night (A) Regularity of sleep (R) and Snoring (S)
BEDS	Behavioral Evaluation of Disorders of Sleep
BISQ	Brief Infant Sleep Questionnaire
BRIAN-K	Biological Rhythm Interview of Assessment in Neuropsychiatry – Kids
CAS-15	Clinical Assessment Score-15
CBCL	Child Behavior Checklist sleep items
CCTQ	Children's ChronoType Questionnaire
CRSP	Children's Report of Sleep Patterns
CRSP-S	Children's Report of Sleep Patterns – Sleepiness Scale
CSAQ	Children's Sleep Assessment Questionnaire
CSHQ	Children's Sleep Habits Questionnaire
CSM	Composite Scale of Morningness
CSRQ	Chronic Sleep Reduction Questionnaire
CSWS	Children's Sleep-Wake Scale
DBAS	dysfunctional beliefs and attitudes about sleep scale
ESS-CHAD	Epworth Sleepiness Scale for Children and Adolescents
FoSI	Fear of Sleep Inventory
I SLEEPY	I SLEEPY, short pediatric sleep apnea questionnaire
IF SLEEPY	IF SLEEPY, short pediatric sleep apnea questionnaire
I'M SLEEPY	I'M SLEEPY, short pediatric sleep apnea questionnaire
ISI	Insomnia Severity Index
JSQ	Japanese Sleep Questionnaire
LSTCHQ	Sleep Length and Television and Computer Habits of Swedish School-Age Children
MCTQ	Munich ChronoType Questionnaire
MEQ	Morningness-Eveningness Questionnaire
aMEQ-R	reduced Morningness-Eveningness Questionnaire
MESC	Morningness–Eveningness Scale for Children
MESSi	Morningness–Eveningness Stability Scale improved
My Sleep and I	
My children's sleep	
NARQoL-21	narcolepsy-specific HrQoL self-report questionnaire
NSD	nighttime sleep diary
NSS	Narcolepsy Severity Scale (Chinese)
OSA Screening Questionnaire	Obstructive Sleep Apnea Screening Questionnaire
OSA-18 Questionnaire	Obstructive Sleep Apnea Questionnaire
OSD-6 QoLQuestionnaire	obstructive-sleep-disorders-6-survey
oSDB and AT	Obstructive Sleep-Disordered Breathing and Adenotonsillectomy Knowledge Scale for Parents
OSPQ	omnibus sleep problems questionnaire
PADSS	Paris Arousal Disorders Severity Scale
PDSS	Pediatric Daytime Sleepiness Scale
Pediatric Sleep CGIs	Pediatric Sleep Clinical Global Impressions Scale
PedsQL	Pediatric Quality of Life (PedsQL) Multidimensional Fatigue Scale
PISI	Pediatric Insomnia Severity Index
PNSSS	Parent Newborn Sleep Safety Survey
PosaST	pediatricobstructive sleep apnea screening tool
PPPS	Puberty and Phase Preference Scale (also cited as Morningness Eveningness Scale)
P-RLS-SS	Pediatric Restless Legs Syndrome Severity Scale
PROMIS	Patient-Reported Outcomes Measurement Information System (PROMIS) Sleep Disturbance and Sleep-Related Impairment item banks
PSIS	Parent-Child Sleep Interactions Scale
PSQ	Pediatric Sleep Questionnaire
PSQI	Pittsburgh Sleep Quality Index
RLS	Restless legs syndrome
SDIS	Sleep Disorders Inventory for Students
SDPC	Sleep Disturbances in Pediatric Cancer
SDSC	Sleep Disturbance Scale for Children
SDSC*	Sleep Disturbances Scale for School-age Children
SHI	Sleep Hygiene Index
SHIP	Sleep Hygiene Inventory for Pediatrics
Sleep Bruxism	parental-reported sleep bruxism
SNAKE	a questionnaire on sleep disturbances in children with severe psychomotor impairment (Schlaffragebogen für Kinder mit Neurologischen und Anderen Komplexen Erkrankungen)
SQI	Sleep Quality Index
SQ–SP	Sleep Questionnaire developed by Simonds and Parraga
SQS-SVQ	sleep quality scale and sleep variables questionnaire
SRSQ	Sleep Reduction Screening Questionnaire
SSR	Sleep Self-Report
SSSQ	simple self-report sleep questionnaire
STBUR	(Snoring, Trouble Breathing, Un-Refreshed questionnaire
STQ	Sleep Timing Questionnaire
The Children's Sleep Comic	
TuCASA	Tucson Children's Assessment of Sleep Apnea Study
YSIS	Youth Self−Rating Insomnia Scale
